# Influence of Interlayer Cation Ordering on Na Transport in P2-Type Na_0.67–*x*_Li_*y*_ Ni_0.33–*z*_Mn_0.67+*z*_O_2_ for Sodium-Ion Batteries

**DOI:** 10.1021/jacs.4c00869

**Published:** 2024-05-02

**Authors:** Eric Gabriel, Zishen Wang, Vibhu Vardhan Singh, Kincaid Graff, Jue Liu, Cyrus Koroni, Dewen Hou, Darin Schwartz, Cheng Li, Juejing Liu, Xiaofeng Guo, Naresh C. Osti, Shyue Ping Ong, Hui Xiong

**Affiliations:** † Micron School of Materials Science and Engineering, Boise State University, Boise, Idaho 83725, United States; ‡ Department of NanoEngineering, University of California, San Diego, La Jolla, California 92093, United States; § Center for Nanoscale Materials, Argonne National Laboratory, Argonne, Illinois 60439, United States; ∥ Neutron Scattering Division, Oak Ridge National Laboratory, Oak Ridge, Tennessee 37830, United States; ⊥ Department of Geosciences, Boise State University, Boise, Idaho 83725, United States; # Department of Chemistry, Washington State University, Pullman, Washington 99164, United States

## Abstract

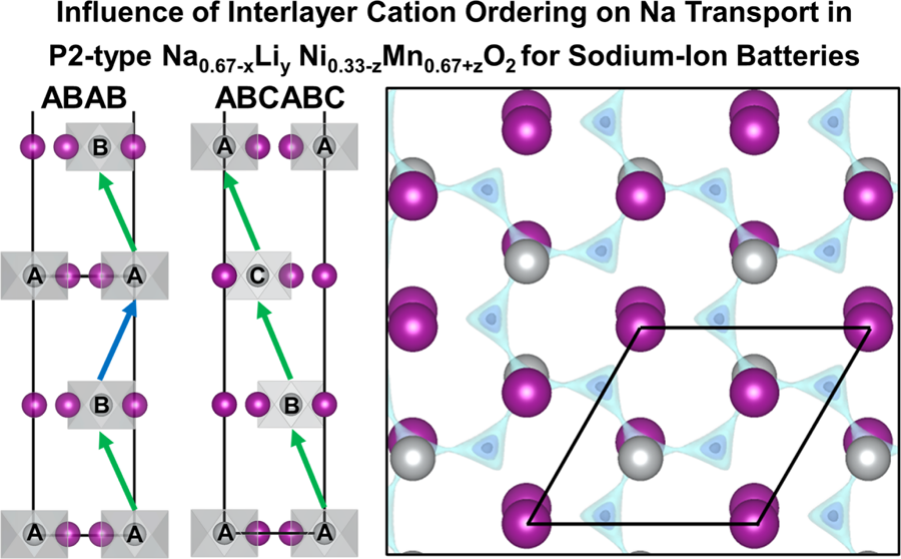

P2-type Na_2/3_Ni_1/3_Mn_2/3_O_2_ (PNNMO) has been extensively studied because of its desirable electrochemical properties as a positive electrode for sodium-ion batteries. PNNMO exhibits intralayer transition-metal ordering of Ni and Mn and intralayer Na^+^/vacancy ordering. The Na^+^/vacancy ordering is often considered a major impediment to fast Na^+^ transport and can be affected by transition-metal ordering. We show by neutron/X-ray diffraction and density functional theory (DFT) calculations that Li doping (Na_2/3_Li_0.05_Ni_1/3_Mn_2/3_O_2_, LFN5) promotes ABC-type interplanar Ni/Mn ordering without disrupting the Na^+^/vacancy ordering and creates low-energy Li–Mn-coordinated diffusion pathways. A structure model is developed to quantitatively identify both the intralayer cation mixing and interlayer cationic stacking fault densities. Quasielastic neutron scattering reveals that the Na^+^ diffusivity in LFN5 is enhanced by an order of magnitude over PNNMO, increasing its capacity at a high current. Na_2/3_Ni_1/4_Mn_3/4_O_2_ (NM13) lacks Na^+^/vacancy ordering but has diffusivity comparable to that of LFN5. However, NM13 has the smallest capacity at a high current. The high site energy of Mn–Mn-coordinated Na compared to that of Ni–Mn and higher density of Mn–Mn-coordinated Na^+^ sites in NM13 disrupts the connectivity of low-energy Ni–Mn-coordinated diffusion pathways. These results suggest that the interlayer ordering can be tuned through the control of composition, which has an equal or greater impact on Na^+^ diffusion than the Na^+^/vacancy ordering.

## 1 Introduction

Sodium-ion batteries (SIBs) are a promising electrochemical energy storage technology because they are readily prepared from earth-abundant elements. Among the positive electrode materials for SIBs, the layered transition-metal oxides (LTMOs) are especially attractive due to their dense structure and high redox potential compared to Prussian Blue analogues,^1^ and polyanionic compounds.^2−4^ LTMOs can combine multiple transition-metal elements and take several polymorphs (O3, P2, P3, and O2 in the notation of Delmas),^5^ resulting in highly tunable properties. The LTMO structures are distinguished by the sodium site geometry (octahedral or prismatic) and the number of TMO_2_ layers in the unit cell. The P2 structure (space group *P*6_3_/*mmc*) is desirable for its fast Na^+^ transport properties, which arise from the low diffusion barrier between adjacent prismatic sites in the Na^+^ layer. The P2 structure has two distinct Na^+^ sites: one that is face-sharing with the transition-metal (TM = Ni, Co, Fe, Mn) ions in the adjacent TM layers (TM–Na–TM) and another that is edge-sharing, coordinated by tetrahedral vacancies in the adjacent TM layers (V–Na–V). In multicomponent P2-type LTMOs, the composition of the TM ions in the face-sharing site is known to be a critical factor for the Na^+^ diffusion kinetics.^6^ P2-type Na_2/3_Ni_1/3_Mn_2/3_O_2_ (PNNMO) has received great attention because it exhibits good stability over many cycles and is air-stable.^7^ The compositional ratio of 1:2 and the difference in size and the valence between Ni^2+^ and Mn^4+^ promote a honeycomb-like ordering of the TM ions within each TM layer.^8,9^

Systematic studies on the structure of several A_2/3_[M′_1/3_^2+^M_2/3_^4+^]O_2_ compounds (A = Na, Li; M′= Ni, Mg; M = Mn, Ti) were conducted by Dahn and co-workers in the early 2000s and several similar systems more recently.^8,10−14^ The Na-based LTMOs with Ni_1/3_Mn_2/3_ or Mg_1/3_Mn_2/3_ have strong intralayer M′/M ordering,^10^ while Ni_1/3_Ti_2/3_, Fe_1/3_Mn_2/3_, and Co_1/3_Mn_2/3_ have weak short-range ordering or no ordering whatsoever.^8,10,13,14^ Other than the intralayer (within layers) ordering of M′ and M ions, the composition also influences the interlayer (between layers) M′/M ordering. In adjacent TM layers for Ni_1/3_Mn_2/3_, Ni stacks alternately between two sites (ABAB), while in Mg_1/3_Mn_2/3_, the Mg ions stack directly on top of each other (AAAA).^10^ It was also found that both regions of correlated and uncorrelated interlayer cation arrangements were present for the P3 polymorph of Na_2/3_Ni_1/3_Mn_2/3_O_2_.^8^ These results suggest that the interlayer cation ordering is a tunable property that composition and synthesis conditions can control. Since the interlayer ordering will dictate which TM–Na–TM configurations are present (i.e., Ni–Na–Mn, Ni–Na–Ni, and Mn–Na–Mn) and their relative amounts, this could be a powerful avenue to influence the Na^+^ diffusion kinetics that depend directly on these configurations. Additionally, the in-plane electrostatic repulsion between Na^+^ and their interaction with the ordered TM layer gives rise to the “large zigzag” (LZZ) Na^+^/vacancy ordering in PNNMO and similar structures.^9,15−17^ Many efforts, mostly by cationic substitutions or doping, have been made to disrupt the Na^+^/vacancy ordering with the belief that it is detrimental to the Na^+^ diffusion.^9,17−20^

Here, we show by neutron powder diffraction (NPD) and X-ray diffraction (XRD) that the interplanar Ni/Mn ordering can be modified by controlled Li doping without disrupting the Na^+^/vacancy ordering. Density functional theory (DFT) calculations show that Li doping promotes an ABCABC-type interlayer Ni/Mn ordering and generates low-energy Li–Mn-coordinated Na sites. Quasielastic neutron scattering (QENS) provides the Na^+^ diffusion mechanism, revealing that Na^+^ diffusivity in the Li-doped structure (with Na^+^/vacancy ordering) is enhanced by an order of magnitude and is comparably fast to Na_2/3_Ni_1/4_Mn_3/4_O_2_ (without Na^+^/vacancy ordering). The Li-doped material (Na_2/3_Li_0.05_Ni_1/3_Mn_2/3_O_2_, LFN5) delivers the largest specific capacity at current rates up to 450 mA g^–1^ compared to PNNMO as a result of lower interlayer Na^+^–Na^+^ repulsion. However, the increased Mn content in Na_2/3_Ni_1/4_Mn_3/4_O_2_ (NM13) disrupts the connectivity of the low-energy Ni–Mn-coordinated network of Na^+^ diffusion paths. This disruption limits the macroscopic Na^+^ transport and results in a lower capacity for NM13 than PNNMO and LFN5 above 180 mA g^–1^ current rate. These results suggest that the interlayer ordering can be tuned through the control of composition and that the interlayer ordering may have an equal or greater impact on Na^+^ diffusion than the Na^+^/vacancy ordering.

## 2 Results and Discussion

### 2.1 Interlayer Transition-Metal and Na^+^/Vacancy Ordering (SXRD and NPD)

#### 2.1.1 Interlayer Transition-Metal Ordering

A series of P2-type Na_0.67–*x*_Li_*y*_Ni_0.33–*z*_Mn_0.67+*z*_O_2_ materials (*x* = 0, 0.10; *y* = 0.0, 0.05, 0.10, 0.20; *z* = 0, 0.08) were prepared by the solid-state reaction of coprecipitated transition-metal hydroxides and sodium/lithium carbonates (Table 1). Select samples were measured by inductively coupled plasma mass spectroscopy (ICP-MS), and the data agree with the designed compositions (Table S1). Pristine PNNMO (*x* = *y* = *z* = 0.0) can be doped with lithium in a controlled manner to tune the site Li occupies on either the TM or Na layer.^21^

**Table 1:** Designed Composition and Sample Labeling Scheme of the Prepared Materials

sample name	*x*	*y*	*z*	overall formula
PNNMO	0.0	0.0	0.0	Na_0.67_Ni_0.33_Mn_0.67_O_2_
NM13	0.0	0.0	0.08	Na_0.67_Ni_0.25_Mn_0.75_O_2_
LFN5	0.0	0.05	0.0	Na_0.67_Li_0.05_Ni_0.33_Mn_0.67_O_2_
LFN10	0.0	0.10	0.0	Na_0.67_Li_0.10_Ni_0.33_Mn_0.67_O_2_
LFN20	0.0	0.20	0.0	Na_0.67_Li_0.20_Ni_0.33_Mn_0.67_O_2_
LSN10	0.10	0.10	0.0	Na_0.57_Li_0.10_Ni_0.33_Mn_0.67_O_2_

**Figure 1:**
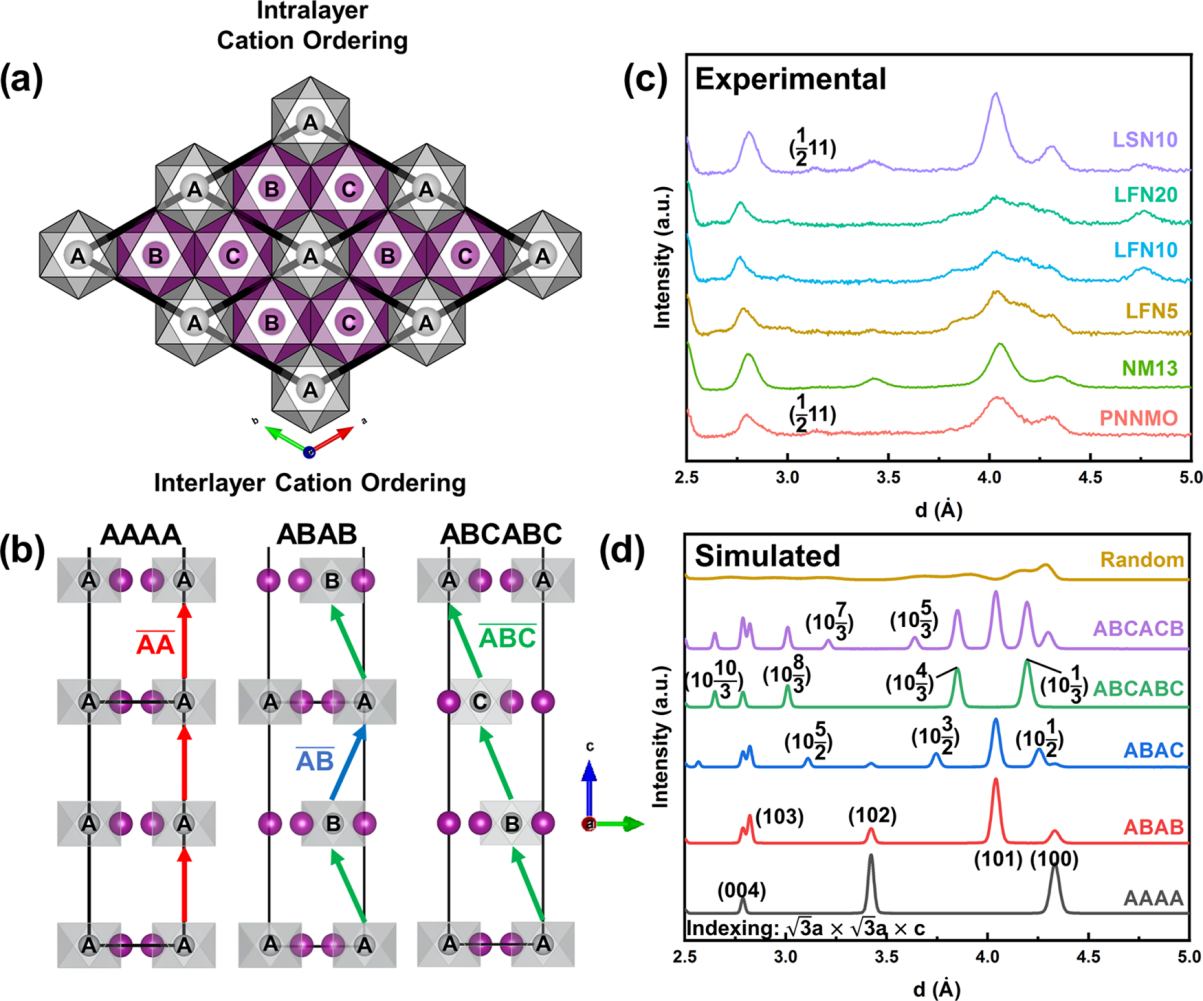
(a) Top-down view of the intralayer honeycomb ordering in Na_2/3_Ni_1/3_Mn_2/3_O_2_ with distinct A, B, and C sites labeled. The unit cell edges are indicated by black lines. (b) Schematic of AAAA, ABAB, and ABCABC interlayer ordering schemes and associated interlayer vectors. Only half of the ABCABC-ordered unit cell is shown for the sake of visual simplicity. (c) Experimental neutron diffraction patterns and (d) simulated neutron diffraction of different interlayer arrangements of TM ions.

If sodium is substituted for lithium (*x* = *y*) as in LSN10, Li will predominately occupy sites on the sodium layer.^21^ In contrast, if excess Li is added without modifying the Na content as in the LFN5, LFN10, and LFN20 samples, Li will predominately occupy sites in the transition-metal layer.^21^ It has also been shown that with a slight modification of the Ni/Mn ratio, from 1:2 in PNNMO to 1:3 as in NM13, the Na^+^/vacancy ordering can be disrupted.^9^ These modifications to the structure have different influences on the interlayer TM ordering due to varying electrostatic interactions between the honeycomb-ordered layers. To examine the interlayer transition-metal (Ni and Mn) ordering, we conducted an NPD experiment. Ni and Mn are not readily distinguishable by X-ray diffraction (XRD) due to their similar electron densities, while the different coherent neutron scattering lengths of Ni and Mn (10.3 fm vs −3.73 fm, respectively) provide a good contrast between them. The √3*a* × √3*a* supercell needed to explain the intraplanar cation ordering in the TM layer has 3 unique TM positions in each plane that are assigned as A, B, and C at relative positions (0, 0, *z*), (1/3, 2/3, *z*), and (2/3, 1/3, *z*) of the expanded cell, respectively, similarly to previous reports.^22,23^ Ni occupies one of the three sites, while Mn occupies the other two to form the honeycomb-like arrangement (Figure 1a). Note that this labeling of the TM positions is not related to the oxygen stacking sequence as described by Delmas, which remains fixed in the typical alternating AB BA sequence where all transition metals sit on the C site of the TM-disordered P2 unit cell (P6_3_/*mmc*).^5^ This discussion is only concerned with modifications of the ordering of the transition metals while the oxygen stacking sequence is unperturbed, so for simplicity of notation, these labels (A, B, C) refer to the TM-ordered √3*a* × √3*a* superlattice positions.

The relative arrangement of Ni between layers allows for multiple distinct possible interplanar orderings (Figure 1b). The fixed 2-layer oxygen stacking sequence of the P2 structure will dictate that TM stacking sequence be composed by an even (2*n* = 2, 4, 6, ...) number of layers. The interplanar configuration of the honeycomb layers will have a significant effect on the diffraction pattern, as shown by the experimental and simulated NPD patterns for different interplanar orderings (Figure 1c,d). Numerous (10*l*) peaks appear in the simulated patterns that can be indexed with fractional *l* values corresponding to the 4/6-layer larger unit cells, especially for those with less symmetric sequences (e.g., ABAC).

**Figure 2:**
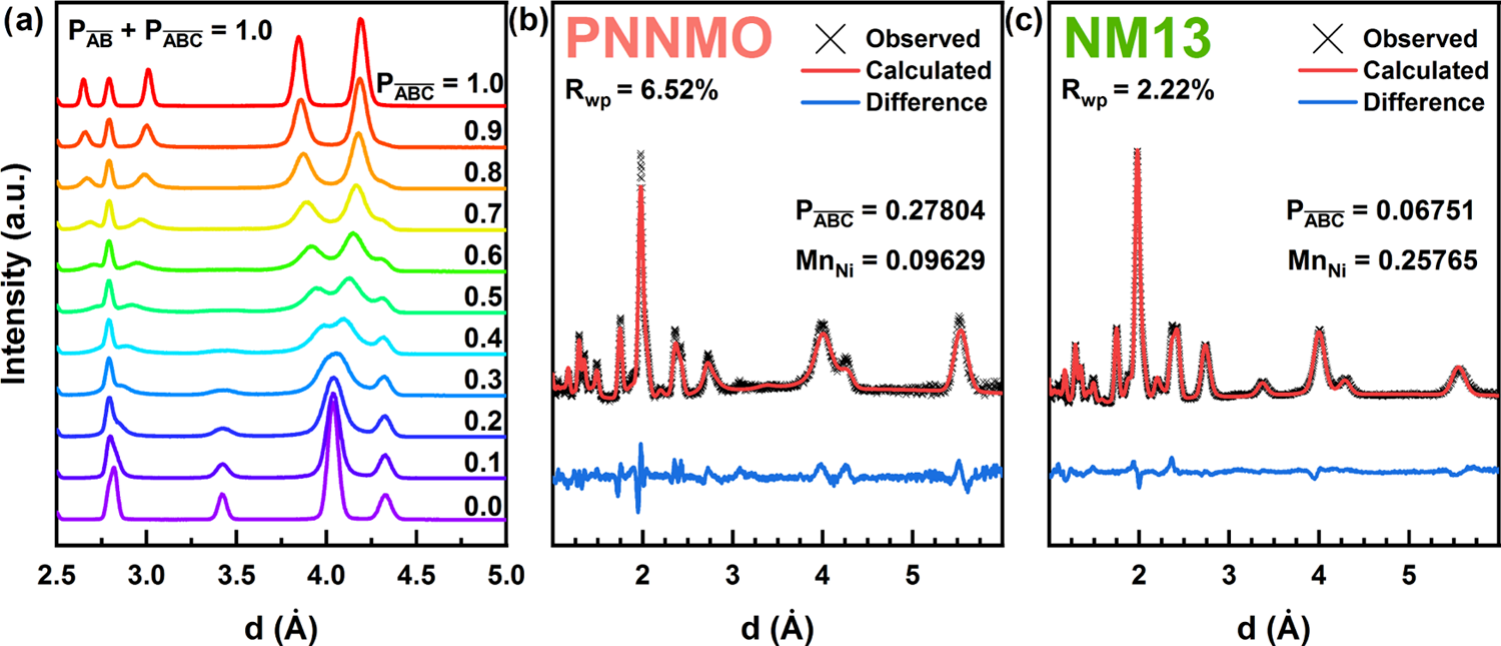
(a) Simulated diffraction patterns of the intermediate interlayer orderings between the ABAB- and ABCABC-type orderings (). (b, c) Refined neutron diffraction patterns of PNNMO and NM13, respectively.

NPD of the undoped sample (PNNMO) appears most similar to the ABAB interplanar arrangement, consistent with previous reports.^9,10,23^ The LZZ ordering (indexed as (1/2 1 1)) is also evident in the NPD patterns for PNNMO, LFN5, and LSN10. A notable difference from previous reports is the absence of the (102) peak at ∼3.42 Å.^23,24^ The difference from previous reports could possibly arise from differences in the processing conditions affecting the crystallinity. Specifically, the calcination temperature, time, and quenching procedure could allow for differences in the degree of crystallinity. To explain the different (102) peak intensities, two hypotheses are considered. First, site mixing between the Ni and Mn could alter the structure factor to selectively reduce this peak; or second, the observed TM stacking arrangement contains a significant degree of faults that deviate from the ideal ABAB sequence. The first hypothesis cannot fully explain the suppressed (102) peak intensity based on simulations (using the same instrument parameters as the experimental measurement) of different modes of Ni/Mn site mixing (Figure S1), where neither uniform mixing of Ni onto both Mn sites (i.e., A onto B and C) nor between Ni and individual Mn sites (i.e., A onto B, or A onto C) results in the fully suppressed intensity of the (102) peak. Alternatively, some degree of disorder in the interlayer cation stacking sequence could result in suppression or distortion of specific peaks. The various interlayer stacking sequences can be simulated from a probabilistic view using FAULTS software (based on DIFFaX).^25,26^ Any arbitrary cation stacking sequence can be constructed given the defined geometry of a cation-ordered layer and a set of 3 unique stacking vectors (Figure 1b, , , and  where the fractional *z*-coordinate is relative to the regular P2 unit cell). The probability of some stacking vector *R̅* is *P*_*R̅*_, subject to the constraint that the sum of all stacking vector probabilities for any given layer is 1 (∑_*R̅*_*P*_*R̅*_ = 1). The ABCABC structure is generated from repeated  vectors () and the AA structure from repeated  vectors (). The ABAB structure is generated from an alternating sequence of , , , , ..., (one layer with  followed by ). The intermediate structures between the ABAB and ABCABC structures can then be seen as arising from the different probability of the stacking vector that follows the  vector. Simulation with alternating  and then  (the end members of which are the ABAB or ABCABC stacking sequences) begins to reproduce the suppressed intensity of the (102) peak in the PNNMO sample (Figure 2a) around . Refinement of the faulted structure (Figure 2b) indicates that PNNMO exhibits a combination of both intralayer TM mixing (Mn_Ni_ = 9.6 ± 0.47%) and interlayer cationic (Ni and Mn) stacking fault disorder () while having an ABAB-type structure on average. The detailed refinement results for PNNMO are given in Table S2.

NM13 appears to be well crystallized in the ABAB sequence based on its close matching to the simulated patterns. According to the refinement of the same model, NM13 has a much smaller degree of interlayer disorder () compared to PNNMO and close to the expected degree of cation mixing (Mn_Ni_ = 25.8 ± 0.47% actual, 25% expected) according to the lower Ni/Mn ratio. For both samples, the faulted model provides a significantly superior fit (*R*_wp_ = 6.48 and 2.22% for PNNMO and NM13, respectively) compared to the traditional Rietveld refinement (*R*_wp_ = 9.25 and 2.68%) with equivalent constraints (Table S3).^27^ However, the LFN samples match none of the 2–6-layer sequences closely. Instead, features of several distinct orderings appear to be present.

**Figure 3:**
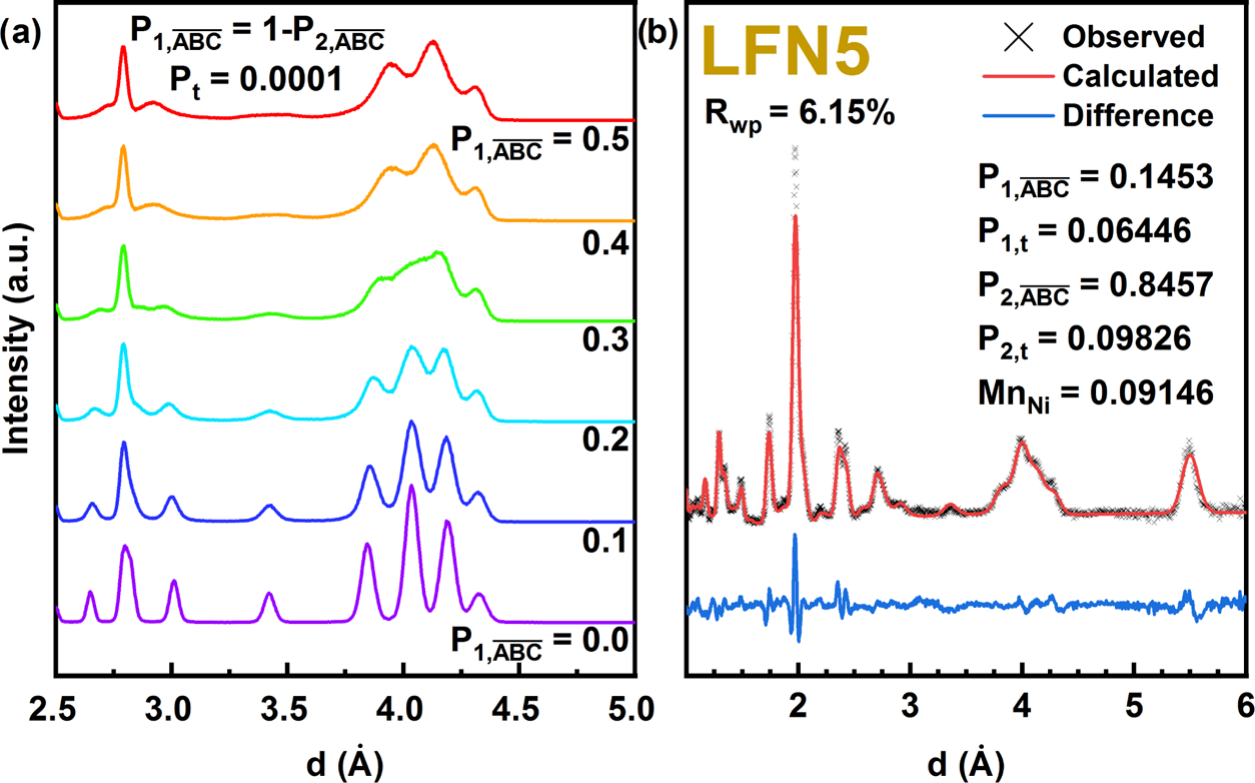
(a) Simulated neutron diffraction patterns of the structure with ABAB- and ABCABC-like clusters, with varied degrees of internal faulting of the opposite type. (b) Refinement of LFN5 according to a clustered faulting model.

In order to consider whether the observed patterns could arise from a combination of differently ordered domains, such as clusters of ABAB- and ABCABC-stacked regions, additional probability terms are incorporated into the simulated stacking sequence model. Each clustered domain *i* is assigned its own probabilities  and an additional probability P_*i*,t_ to describe the frequency that the stacking sequence changes to the type in the alternative domain. The probabilities within a domain *i* are then constrained such that . The first term includes the probabilities of the in-cluster stacking vectors, while the second represents the probability of a vector between clusters. The values of *P_i,t_* then correspond to the average number of layers (*P*_*i*,*t*_^–1^) between clustered domains (which affects peak width; Figure S2), while the ratio of *P_i,t_*^–1^ to the sum of *P_i,t_*^–1^ for each domain (over *i*) determines the layer fraction, *x*_*i*_ (; Figure S3). This model simultaneously captures the presence of distinct types of clusters and the cation stacking-faulted nature of each cluster. These distinct clusters can manifest in the diffraction pattern clearly for clusters with an average number of layers as few as 10 (Figure S2). Simulated structures with 10,000 layer clusters (*P*_1,*t*_ = *P*_2,*t*_ = 0.0001) of ABAB- and ABCABC-like domains, each with faulting of the other type internally (like in Figure 3), are shown in Figure 3a. The  simulation (purple) represents a 50–50 mixture of ideally ordered ABCABC and ABAB domains, while the  simulation (red) is a combination of equivalent faulted ABC/AB domains, exactly like that in the  structure shown in Figure 2. The clustered mixture is significantly closer to the experimental LFN5 structure than any single-ordered stacking sequence (Figure 3b). Refinement of the clustered stacking model provides reasonable agreement with the experimental data (Table S2, *R*_wp_ = 6.2%) and provides a significant^27^ improvement compared to a traditional Rietveld refinement with a combination of ideally ordered ABAB and ABCABC phases (Table S3, *R*_wp_ = 7.6%) with equivalent constraints applied. The relatively large values of *P*_1,*t*_ and *P*_2,*t*_ (0.06446 and 0.09826, respectively) suggest that the different interlayer orderings are only coherent over short ranges of 22–35 nm, which is determined from P_*i*,t_^–1^ multiplied by the distance between layers (see the note in the Methods section of the Supporting Information regarding the refinement of *P_i,t_*). The preference to incorporate clusters of ABC-type domains in the Li-doped samples could be explained by the low valence of Li^+^ modifying the interlayer electrostatic repulsion. In the ABAB stacking, there will be channels of Mn^4+^–Mn^4+^–Mn^4+^ contacts extended along the *c*-axis (Figure 1b) that generate strong interlayer repulsion. In contrast, the ABCABC sequence has only Ni^2+^–Mn^4+^–Mn^4+^ sequences (Figure 1b), which may reduce the degree of interlayer electrostatic repulsion. The incorporation of Li^+^ onto the TM layer could promote similar Li^+^–Mn^4+^–Mn^4+^ configurations to further reduce the interlayer repulsion. This is consistent with the reduction of interlayer spacing in LFN5 compared to PNNMO previously determined by XRD.^21^

Overall, lithium filling (LFN samples, Na + Li > 0.67) doping promotes ABCABC ordering, while lithium substitution (LSN10, Na + Li = 0.67) and altered Ni/Mn ratio (as in NM13) maintain or promote the ABAB ordering. Control over these compositional and processing variables is an avenue to control the interlayer TM ordering in these samples.

#### 2.1.2 Na^+^/Vacancy Ordering

**Figure 4:**
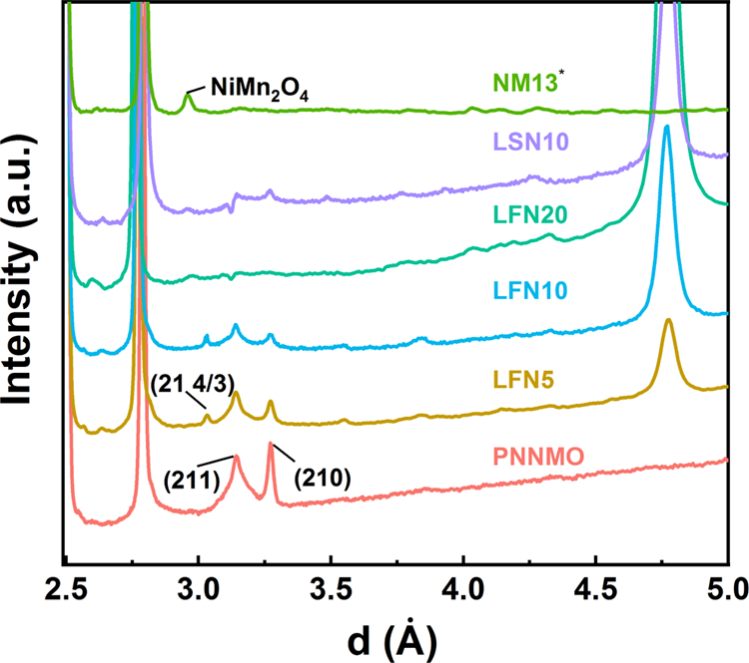
sXRD showing the region associated with Na^+^/vacancy ordering. All data other than NM13 are plotted without background subtraction. *NM13 was measured on a different instrument with a strongly sloping background that was subtracted for easier visual comparison. The small features other than the indicated NiMn_2_O_4_ peak are artificial residuals from background subtraction with a Chebyshev polynomial function. The raw NM13 sXRD pattern is presented in Figure S4.

The similar scattering power of Ni and Mn for X-rays combined with the high brilliance of synchrotron X-ray sources makes synchrotron XRD (sXRD) particularly well-suited to studying the Na^+^/vacancy ordering. To examine the influence of the different compositions on Na^+^/vacancy ordering, we conducted sXRD (Figure 4).

PNNMO, LFN5, and LFN10 show clear superstructure peaks at ∼3.14 and 3.27 Å that are ascribed to the LZZ Na^+^/vacancy ordering, which correspond to the {210} and {211} reflections in the expanded 2√3*a* × 2√3*a* × *c* unit cell. The {211} reflection is notably broader than {210}, suggesting that the intraplanar Na^+^/vacancy ordering is coherent over longer lengths than the interplanar ordering. LSN10 has some indication of the {210} and {211} peaks, but their asymmetric shape may suggest interlayer Na^+^/vacancy disorder, while the clear {210} peak in the neutron diffraction pattern confirms the intralayer ordering in LSN10. The LFN5 and LFN10 samples have an additional peak at ∼3.03 Å that can be indexed as (21 4/3), which suggests a 3*c* superlattice. A 6-layer structure (3*c*) agrees with the neutron diffraction data that indicates a significant fraction of ABCABC-ordered regions, suggesting that the interlayer Na^+^/vacancy ordering is coupled with the interlayer TM ordering. Curiously, the (21 4/3) peak is present in some previous reports for PNNMO^28^ but not others,^9,23^ which suggests that different interlayer Na^+^ orderings may be possible without doping depending on the degree of interlayer TM ordering.

LFN20 and NM13 have no distinct peaks associated with Na^+^/vacancy ordering. The sXRD patterns of Li-doped samples generally have weak indications of the diffraction features associated with the TM layer ordering as described in relation to the NPD results previously. The results of the structural characterizations are summarized in Table 2.

**Table 2:** Summary of the Cation Ordering Characteristics of Each Sample Determined by Combined XRD and NPD Analyses

sample	TM interlayer ordering	Na^+^/vacancy ordering
PNNMO	ABAB	yes
NM13	ABAB	no
LFN5	ABAB + ABC	yes
LFN10	ABAB + ABC	yes
LFN20	ABAB + ABC	no
LSN10	ABAB	yes

**Figure 5:**
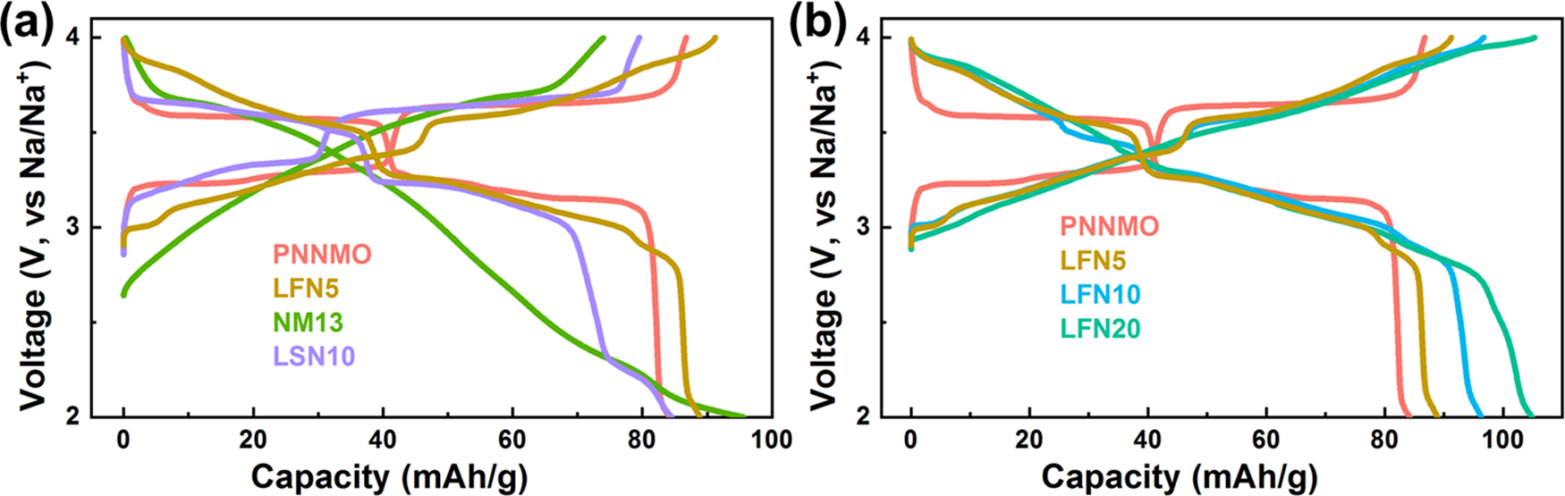
Voltage profile comparison of (a) PNNMO, LFN5, NM13, and LSN10 and (b) PNNMO, LFN5, LFN10, and LFN20.

The shape of the voltage profiles (Figure 5) is directly associated with both the transition-metal and the Na^+^/vacancy ordering present in each sample. PNNMO and LSN10 both have predominantly ABAB-like interlayer transition-metal ordering and Na^+^/vacancy ordering. Correspondingly, PNNMO and LSN10 exhibit well-defined plateaus in their voltage profiles associated with the rearrangements of Na^+^/vacancy ordering. In contrast, NM13 has strong ABAB TM ordering but no Na^+^/vacancy ordering and thus exhibits a mostly sloping voltage profile associated with solid-solution behavior. The samples with a high degree of heterogeneity of the interlayer transition-metal ordering (LFN5, LFN10, and LFN20) have generally smoother voltage profiles. LFN5 and LFN10, which have Na^+^/vacancy ordering, but heterogeneous interlayer TM arrangements, have several clear steps in their voltage profiles separated by regions of solid-solution behavior. In contrast, LFN20 exhibits solid-solution characteristics over the entire first charge due to the lack of Na^+^/vacancy ordering, as in NM13. It has been previously shown for PNNMO,^9^ LSN10,^9^ and NM13^9,21,29^ that there is no layer gliding (i.e., P2 → O2) transition below 4 V so the plateaus must be primarily associated with the Na^+^/vacancy ordering transitions.^28^ Operando sXRD was conducted on LFN10 (Figure S5). The peaks shift during charge/discharge according to the expansion/contraction of the lattice, but no new peaks or peak splitting are observed below 4 V. This suggests that the P2 structure is maintained in LFN10 with no layer gliding phase transformation in the 2–4 V potential range. Similarly to PNNMO, the peaks associated with the LZZ ordering near 3.14 and 3.27 Å reversibly disappear and reappear during charge/discharge (Figure S6) according to the rearrangement of the Na^+^/vacancy ordering.^28^ Similar behavior is expected for LFN5, considering its similarity to PNNMO and LFN10. Additionally, while Li can reversibly migrate between the TM and Na^+^ layers during charge between 2 and 4.4 V,^30^ or irreversibly deintercalated from the Na^+^ layer below 4.1 V,^30^ these processes will not necessarily cause distinct plateaus to appear in the voltage profiles.^30^ Therefore, we conclude that the interaction of the Na^+^/vacancy ordering with the transition-metal ordering is the primary factor that determines the shape of the voltage profile in these samples.

### 2.2 Influence of Interlayer Ordering on Na^+^ Diffusion (QENS, DFT, and Electrochemistry)

#### 2.2.1 Density Functional Theory

**Figure 6:**
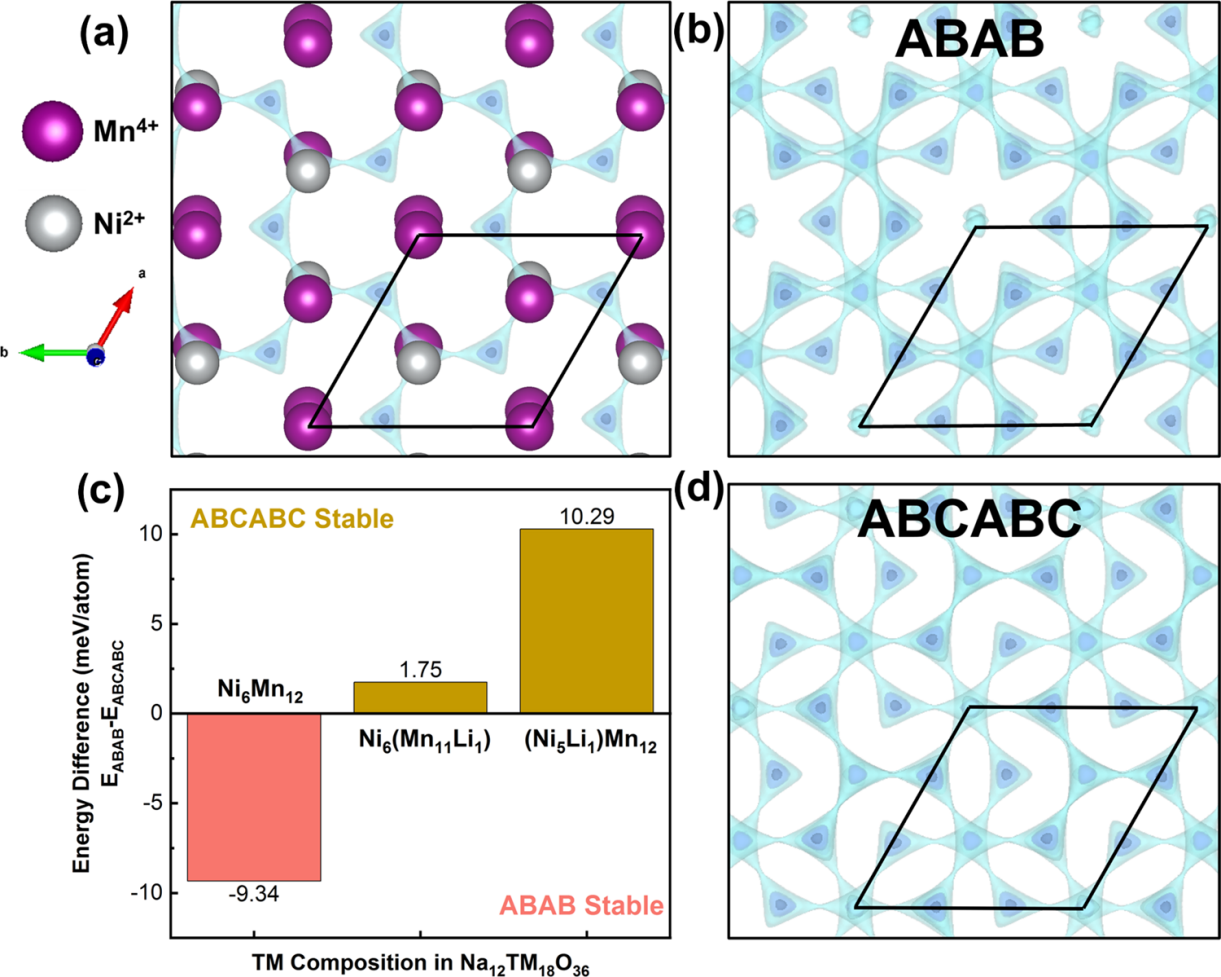
Visualizations of Na^+^ diffusion pathways generated by softBV Bond Valence Pathway Analyzer^31^ and visualized in VESTA^32^ (using equivalent parameters from the refined structure of PNNMO). A darker color of the blue isosurfaces represents a higher density of Na. (a) Na diffusion pathway within a single layer. (b, d) Na^+^ diffusion pathways in the ABAB and ABCABC interlayer transition-metal orderings with 3 layers shown. (c) DFT-calculated energy differences of pristine and Li-exchanged ABAB and ABCABC structures.

In addition to modifying the voltage profiles, the interlayer TM ordering could influence the Na^+^ transport mechanism. The site energies of Mn–Mn-, Ni–Mn-, and Li–Mn-coordinated Na^+^ calculated by density functional theory (DFT) for ABAB-ordered LFN5 (approximated as Na_16_Ni_7_Li_1_Mn_16_O_48_, as detailed in the Supporting Information) are 371, 284, and 60 meV, respectively. These energies suggest that the Na^+^ diffusion path will follow the edge-sharing sites that connect Ni–Mn-coordinated face-sharing sites while avoiding the Mn–Mn-coordinated sites (Figure 6a). In the ABAB interlayer ordering scheme (as in PNNMO and NM13), the channels of Mn^4+^–Mn^4+^–Mn^4+^ along *c* (Figure 1b) will create additional repulsion to Na^+^ ions attempting to diffuse between these sites (Figure 6b). In contrast, the ABCABC stacking (as in LFN5) contains only Ni^2+^–Mn^4+^–Mn^4+^ channels (Figure 1b) that could better distribute the interlayer Na^+^ repulsion (Figure 6d) and facilitate faster Na^+^ transport. The incorporation of lower-valence Li^+^ onto the TM layer in the LFN5 sample further reduces this repulsion, as reflected by the lower site energy of Li–Mn by 200–300 eV compared to other cation pairs. To examine the influence of Li doping and the interlayer ordering scheme and their impact on Na^+^ diffusion, we conducted further DFT calculations. A √3*a* × √3*a* supercell with 6 layers (3*c* in ABAB, 1*c* in ABCABC) was relaxed to determine the relative energies of each structure (Figure 6c).

The calculations reveal that for the pristine ABAB and ABCABC structures, the ABAB structure has a lower energy per atom than ABCABC by 9.34 meV/atom, consistent with the predominately ABAB-ordered experimental structure. After one Ni or Mn atom was exchanged with Li to approximate the composition of the LFN5 sample (i.e., Ni_5/18_Mn_12/18_Li_1/18_ and Ni_6/18_Mn_11/18_Li_1/18_, 1/18 = 0.0555···), the ABCABC structure was found to be lower in energy by 10.29 or 1.75 meV/atom for Ni or Mn substitution, respectively. This aligns with the experimental observation of large ABCABC-ordered domains in the Li-doped samples. Further, the lower energy (by 8.54 meV/atom) of the Ni/Li-exchanged structure compared to that of the Mn/Li-exchanged structure suggests that Li is more likely to occupy the Ni sites in the honeycomb-ordered TM layers.

#### 2.2.2 Quasielastic Neutron Scattering

To correlate the different interlayer orderings, Na^+^/vacancy ordering, and Li doping with the Na^+^ transport mechanism, QENS was performed on the PNNMO, NM13, and LFN5 samples using backscattering silicon spectrometer.^33^ QENS provides the diffusion mechanism by exploring the time and length scales associated with the motion of mobile species like Na^+^. QENS probes shorter time scales (<10^–9^ s) than other techniques that may determine intrinsic diffusivities, such as muon spin resonance and rotation,^34^ or nuclear magnetic resonance that may be complicated in solid, paramagnetic materials.^35,36^ This capability makes QENS well-suited to probe nano- to picosecond dynamics in these P2-type materials. Further, it does not require macroscopic Na^+^ flow between electrodes or (de)sodiation associated with charge or discharge by electrochemical methods such as galvanostatic intermittent titration technique (GITT).^37−39^ The effect on the apparent Na^+^ diffusivity from the other Na^+^ transport processes and varying sodium contents in the electrochemical cell environment is eliminated in the QENS. PNNMO and NM13 have similar interlayer transition-metal ordering (ABAB) but different Na^+^/vacancy ordering. Therefore, a comparison of the QENS data of these samples will identify the influence of the Na^+^/vacancy ordering on the Na^+^ transport mechanism. Similarly, LFN5 has heterogeneous interlayer ordering (ABCABC and ABAB) and Li doping but maintains the Na^+^/vacancy ordering seen in PNNMO. Therefore, a comparison of LFN5 and PNNMO by QENS will identify the influence of the interlayer transition-metal ordering.

We started by collecting the elastically scattered neutron intensity (Figure S7) as a function of temperature to find the temperatures at which the mobility of Na ions falls within the instrument resolution. Analyzable QENS signals were obtained at 450 K and higher temperatures. Four temperature points between 450 and 680 K were selected for the QENS measurements. QENS signals (Figure S8) showed a significant quasielastic broadening as a function of temperatures in all three samples. A similarity of the diffraction patterns of the samples before and after heating and cooling cycles during QENS measurements indicated that the samples remain intact throughout the measurements. Furthermore, the structural integrity of each sample during one heating cycle to 673 K under argon was also verified by ex situ XRD, which is consistent with the results observed in our previous work.^40^

**Figure 7:**
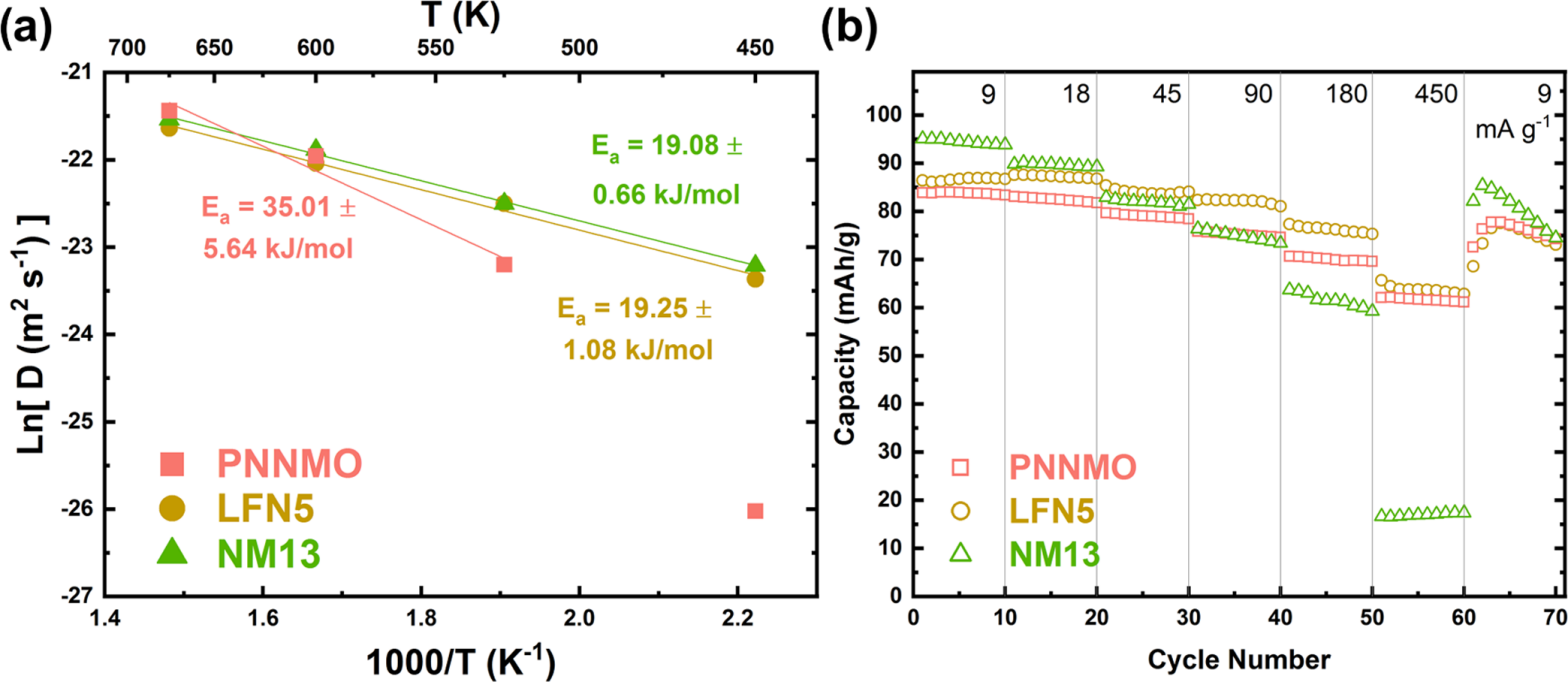
(a) Activation energy for diffusion calculated by Arrhenius fit, with the fit range indicated by the solid lines. (b) Rate performance of PNNMO, LFN5, and NM13 electrodes in CR2032 half-cells vs Na metal with 1 M NaPF_6_ in a propylene carbonate electrolyte.

The *Q*-dependence of half-width at half-maximum (HWHM) of the QENS signal (see the Supporting Information for QENS data analysis details) indicates long-range translational mobility of ions in all samples at all temperatures (Figure S9). At small Q, the slope of the plot provides the diffusivity (D), which shows a strong temperature dependence (Ln(D) in Figure 7a, D in Figure S10).

The diffusivity increases with an increase in temperature for all samples, as expected. However, the diffusivity of PNNMO (0.5 × 10^–11^ m^2^ s^–1^) is notably an order of magnitude smaller at a lower temperature (450 K) than in LFN5 and NM13, which both have similar diffusivities (7.1 × 10^–11^ and 8.3 × 10^–11^ m^2^ s^–1^, respectively). These values are similar in magnitude to those identified by QENS for other P2-type sodium LTMOs,^41,42^ while values determined by electrochemical methods vary by orders of magnitude even for identical composition (Na_2/3_Ni_1/3_Mn_2/3_O_2_, i.e., PNNMO).^28,43−46^ The electrochemically determined values of the Na^+^ diffusivity rely on a number of assumptions or estimations (such as surface area and particle shape/size homogeneity) that may not be satisfied under real conditions,^39^ which highlights the value of QENS as a robust method to probe the atomistic diffusion process. PNNMO shows a large increase of diffusivity after the temperature is increased to 525 K, which is nearly equal to LFN5 and NM13 at 600 and 675 K, respectively. The relatively rapid increase of the PNNMO diffusivity likely results from the melting of the Na^+^/vacancy ordering between 200 and 300 °C (473–573 K). The loss of Na^+^/vacancy ordering can be inferred from the disappearance of the peaks at ∼3.14 and 3.28 Å in the XRD pattern in this temperature range.^17,40^ The activation energy for diffusion can be extracted by linearizing the diffusivity and temperature according to an Arrhenius equation (Figure 7a), where the slope gives the activation energy. The LFN5 and NM13 materials have a similar activation energy of 19.25 and 19.08 kJ/mol (comparable to previous reports of QENS on P2-type Na LTMOs),^41,42^ respectively, while the activation energy for PNNMO is significantly higher. The activation energy (35.01 kJ/mol) for PNNMO is significantly affected by the range of temperatures chosen for the linear fit; the inclusion of the lower temperature data points results in a much higher activation energy (up to 71 kJ/mol between 450 and 525 K) because of the changing slope. The strong temperature dependence of the activation energy suggests that the diffusion mechanism changes for PNNMO during heating, providing direct evidence that thermally disrupting the Na^+^/vacancy ordering can enhance the diffusivity without the need to alter the composition. The similar slopes for NM13 and LFN5 suggest that their diffusion mechanisms do not change between 450 and 675 K.

The difference in the temperature dependence of the diffusivity between LFN5 and PNNMO is interesting because both have Na^+^/vacancy ordering at room temperature. We hypothesize that the superior diffusivity of LFN5 compared to that of PNNMO arises from the different interlayer transition-metal ordering, which does not disrupt the Na^+^/vacancy ordering (as in NM13) and the presence of low-energy Li–Mn-coordinated sodium sites. In the ABAB and ABCABC orderings, the interlayer interaction between diffusing Na^+^ ions is altered. In the ABAB structure, the two channels of Ni–Mn–Ni stackings along the *c*-axis facilitate most of the Na^+^ transport, while the Mn–Mn–Mn channel is avoided (Figure 1b). In the ABCABC structure, there are three Ni–Mn–Ni channels and no Mn–Mn–Mn channels. This interlayer arrangement better distributes the interlayer Na–Na repulsion compared with the ABAB ordering (illustrated in Figure 6b,d), ultimately leading to a higher diffusivity in LFN5. The presence of low-valence Li^+^ in LFN5 also reduces the cationic repulsion on the face-sharing sites, although the low Li concentration could limit the benefit of this effect compared to the interlayer ordering that affects all Na ions. While it is clear that the overall P2 structure and Na^+^/vacancy orderings are maintained after the heating and cooling cycles from RT-675 K, we cannot rule out the possibility of changes to the transition-metal ordering that occur during heating. *In situ* observations of the structural changes to the interlayer transition-metal ordering during heating and cooling are of significant interest and will be the subject of future work.

#### 2.2.3 Electrochemical

In order to correlate the Na^+^ diffusion properties determined by QENS and structural differences with the electrochemical rate performance, each material was evaluated for their rate capability at current rates of 9, 18, 45, 90, 180, and 450 mA g^–1^ in the 2–4.0 V window (Figure 7b). The low-rate capacities (9 mA g^–1^, 0.1C) are 83.9, 86.4, and 95.1 mAh g^–1^ for PNNMO, LFN5, and NM13, respectively. At high rate (450 mA g^–1^, 5C), the capacities for PNNMO, LFN5, and NM13 are 62.0, 65.7, and 16.6 mAh g^–1^, respectively, corresponding to 74.0, 76.0, and 17.5% capacity retention compared to the low-rate capacity. The superior capacity retention of LFN5 up to 180 mA g^–1^ (77.3 mAh g^–1^, 89.5%) compared to that of PNNMO (70.7 mAh g^–1^, 84.3%) agrees with the higher diffusivity measured by QENS (7.1 × 10^–11^ and 0.5 × 10^–11^ m^2^ s^–1^ for LFN5 and PNNMO, respectively). However, a further increase of the current to 450 mA g^–1^ eventually causes their capacities to be comparable, reflecting a lower percentage retention for LFN5. This suggests that the Na^+^ diffusivity may not be the limiting factor at a very high current. Similarly, the relatively poor rate performance at 450 mA g^–1^ of NM13 (17.5%) compared to that of LFN5 (76%) despite the higher initial capacity of NM13 is contrary to their similar diffusivities (8.3 × 10^–11^ m^2^ s^–1^ vs 7.1 × 10^–11^ m^2^ s^–1^, respectively) at low temperature (450 K). The significant difference in the transition-metal composition of NM13 may have a greater role in the rate performance than the effect of Na^+^/vacancy ordering on Na^+^ diffusion. Interlayer Mn–Mn configurations have previously been predicted to have a detrimental effect on the Na^+^ diffusion kinetics in P2-type oxides,^6^ which is further supported in PNNMO by the previously discussed site energy calculations. The higher Mn content of NM13 causes 1/4 of the Ni–Mn configurations present in PNNMO to become Mn–Mn configurations, disrupting the connectivity of the low-energy Ni–Mn pathways. At higher rates, the remaining 3/4 of Ni–Mn configurations may become congested, forcing Na^+^ diffusion to slow or occur partially over Mn–Mn sites, ultimately limiting the diffusion under dynamic (de)sodiation (illustrated in Figure S11). The QENS measurement was performed only in the pristine state under static (fixed sodium content) conditions, so the observed diffusivity can still reflect the fast diffusion between the available edge-sharing and Ni–Mn sites. In addition to the sodium diffusivity, other factors may influence the electrochemical rate performance such as the charge-transfer resistance, electronic conductivity, and the development of the cathode-electrolyte interphase. Overall, we find that the interlayer cation ordering is a significant factor in the Na^+^ diffusion mechanism in these P2-type materials. Further investigation of methods to tune the interlayer cation ordering in LTMOs is a promising avenue toward high-power sodium-ion batteries.

## 3 Conclusions

The interlayer cationic stacking sequence in honeycomb-ordered P2-type Na_0.67–*x*_Li_*y*_ Ni_0.33–*z*_Mn_0.67+*z*_O_2_ is explained in AAAA, ABAB, or ABCABC modes. A combination of cationic stacking faults (ABC-like regions) and intralayer cation mixing (Mn on the Ni site) in the ABAB-ordered PNNMO material can explain the disorder features observed in the NPD pattern. Doping Li onto the TM layer (LFNy samples) results in a heterogeneous interlayer Ni/Mn ordering with clustered regions of ABAB and ABCABC ordering. Those clusters are stabilized by the presence of Li on the Ni site, as suggested by DFT computations. The interlayer arrangement of the TMs also influences the interlayer arrangement of the Na^+^/vacancy ordering based on the appearance of additional peaks, corresponding to 6-layer unit cells in the sXRD patterns. The type of interlayer TM ordering in combination with the presence or absence of the Na^+^/vacancy ordering strongly influences the shape of the voltage profile. Voltage plateaus are associated with the interaction of the Na^+^/vacancy ordering with the interlayer TM ordering, where the heterogeneity of the interlayer TM ordering has a smoothing effect on the voltage profile. Similarly, when the Na^+^/vacancy ordering is disrupted, the voltage profiles exhibit sloping solid-solution characteristics instead of plateaus. QENS provided direct evidence that thermally disrupting the Na^+^/vacancy ordering changes the diffusion mechanism in PNNMO. QENS of LFN5 further suggests that Na^+^ diffusion is enhanced (comparable to NM13 with no Na^+^/vacancy order) by the incorporation of ABCABC-ordered domains and Li–Mn-coordinated Na sites, without disturbing the Na^+^/vacancy ordering. This enhanced diffusivity is associated with the heterogeneous interlayer TM ordering, which better distributes the interlayer Na^+^–Na^+^ repulsion in the ABCABC cation ordering than in the ABAB ordering, as well as the low site energy of Li–Mn coordination. Between PNNMO and LFN5, the modified interlayer cation ordering results in enhanced electrochemical rate performance. For NM13, the rate performance is limited by the lower connectivity between Ni–Mn-coordinated face-sharing sites for Na at the higher Mn concentration, despite the Na^+^/vacancy disorder and high local diffusivity between available sites observed from QENS. The various forms of cationic disorder, both intralayer and interlayer, have significant impacts on the Na^+^ diffusion mechanism in the P2-type structures. Synthesis methods to control the interlayer cation ordering can be a valuable direction for the development of high-power electrode materials. The design of the inter- and intralayer cationic ordering will greatly impact the future of practical LTMOs.

## Supporting Information Available

Simulated NPD patterns with different modes of intralayer Ni/Mn mixing, simulated NPD patterns showing the domain size effect, simulated NPD patterns showing the influence of relative domain sizes, sXRD pattern of NM13 without background subtraction, operando sXRD patterns of LFN10, elastic intensity scan of PNNMO and LFN5, representative QENS spectra, Q-dependence of QENS half-width at half-maximum, diffusivity vs inverse temperature for PNNMO, LFN5, and NM13, schematic of the diffusion pathways in NM13, comparison of calculated NPD patterns in the modes used for simulation and refinement, view of the optimized structure for site energy calculations, detailed FAULTS and Rietveld refinement results, and experimental and computational methods (PDF)
