# Supplementary material for: Influence of Interlayer Cation Ordering on Na Transport in P2-Type Na0.67–xLiy Ni0.33–zMn0.67+zO2 for Sodium-Ion Batteries
Source: J Am Chem Soc. 2024 May 2;146(22):15108–18. doi: 10.1021/jacs.4c00869 (PMC11157533; doi:10.1021/jacs.4c00869)
Supplement: Supplementary file 1 — ja4c00869_si_001.pdf [file ja4c00869_si_001.pdf]

Supporting Information

Influence of Interlayer Cation Ordering on Na Transport in P2-type $Na_{0.67-x}Li_y Ni_{0.33-z}Mn_{0.67+z}O_2$ for Sodium-Ion Batteries

Eric Gabriel[a], Zishen Wang[b], Vibhu Vardhan Singh[b], Kincaid Graff[a], Jue Liu[d], Cyrus Koroni[a], Dewen Hou[a,c], Darin Schwartz[e], Cheng Li[d], Juejing Liu[f], Xiaofeng Guo[f], Naresh C. Osti[d,*], Shyue Ping Ong[b,*], Hui Xiong[a,*]

[a]Micron School of Materials Science and Engineering, Boise State University, Boise, ID, 83725, USA

[b]Department of NanoEngineering, University of California, San Diego, La Jolla, CA 92093, USA

[c]Center for Nanoscale Materials, Argonne National Laboratory, Argonne, IL, 60439, USA

[d]Neutron Scattering Division, Oak Ridge National Laboratory, Oak Ridge, Tennessee 37830, USA

[e]Department of Geosciences, Boise State University, Boise, ID, 83725, USA

[f]Department of Chemistry, Washington State University, Pullman, WA, 99164, USA

*Corresponding Author,

Hui Xiong: clairexiong@boisestate.edu,

Shyue Ping Ong: ongsp@ucsd.edu,

Naresh C. Osti: ostinc@ornl.gov

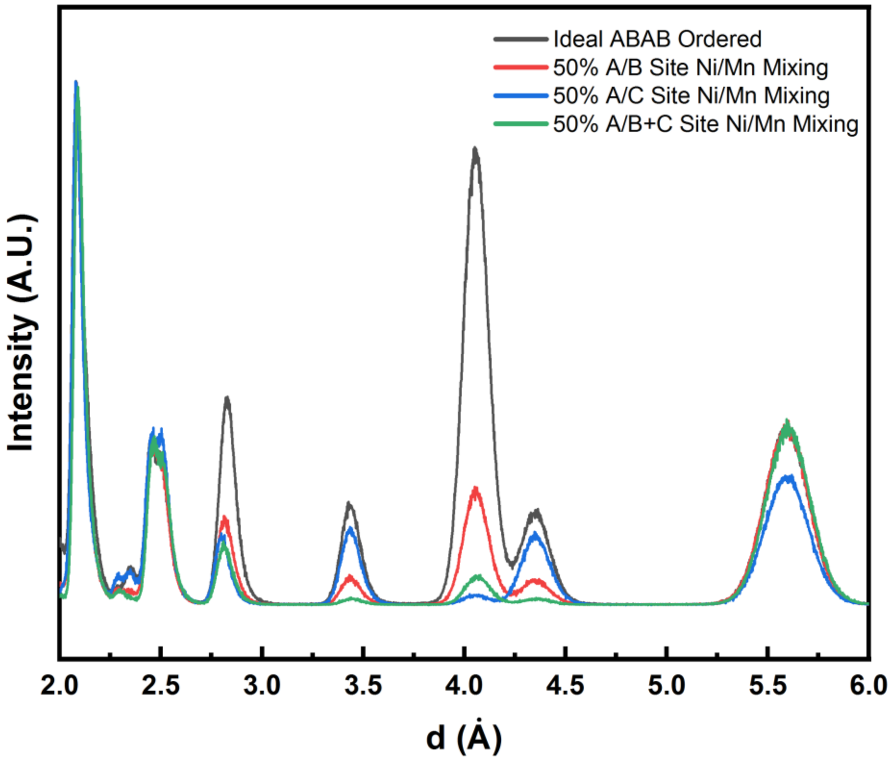

Figure S1. Simulated NPD patterns with different modes of Ni/Mn site mixing. If Ni and Mn are mixed only between two sites (Ni on A, Mn on B or C), (hkl) dependent superlattice peak intensity changes occur, while equal mixing on all sites reduces the intensity of all superlattice peaks similarly. In any case, mixing cannot fully suppress the intensity of the (102) peak at 3.42Å.

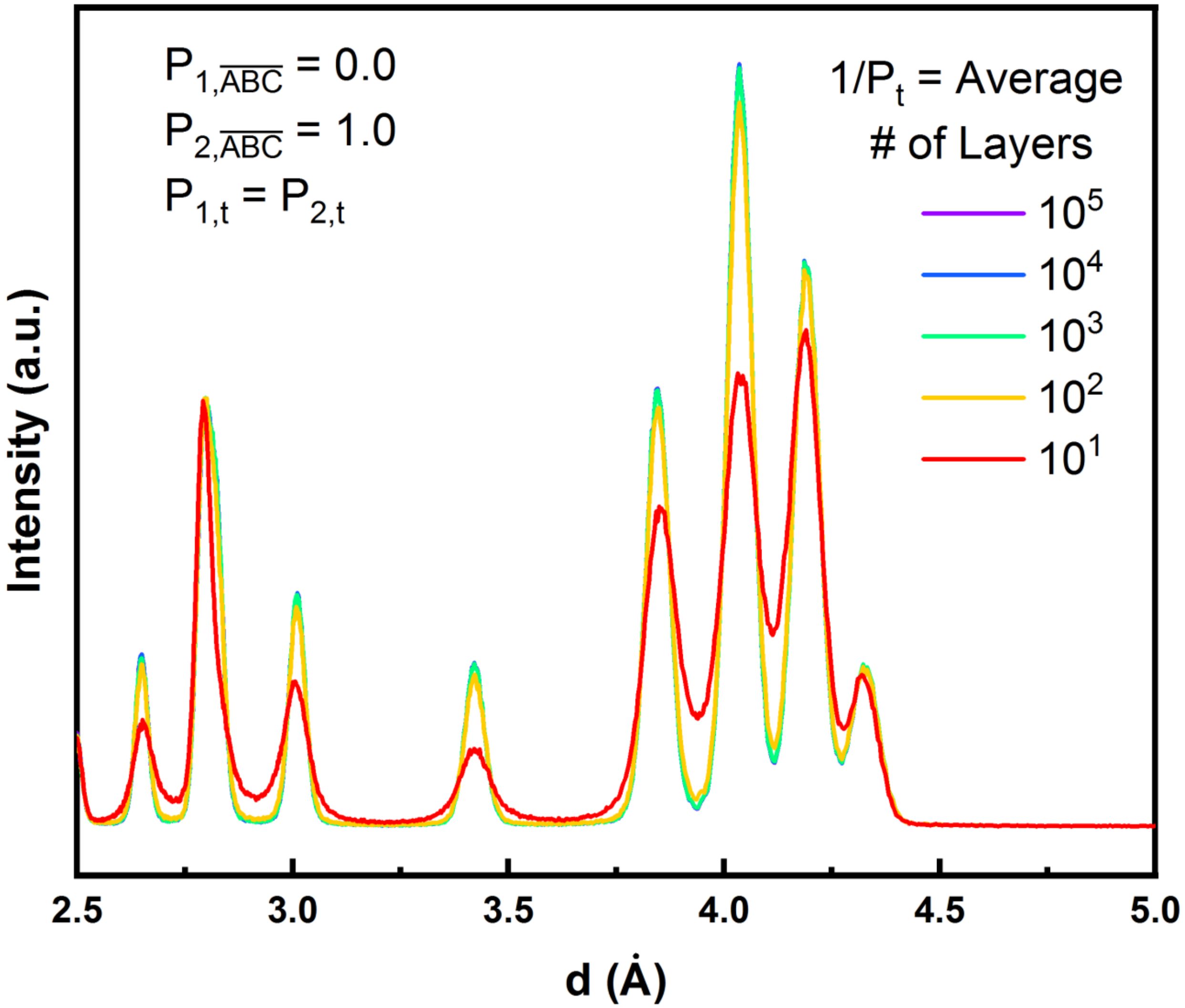

Figure S2: The effect of the domain size ($1/P_t$) for an equal mixture (50% layer fractions) of ideally ordered ABAB and ABCABC domains.

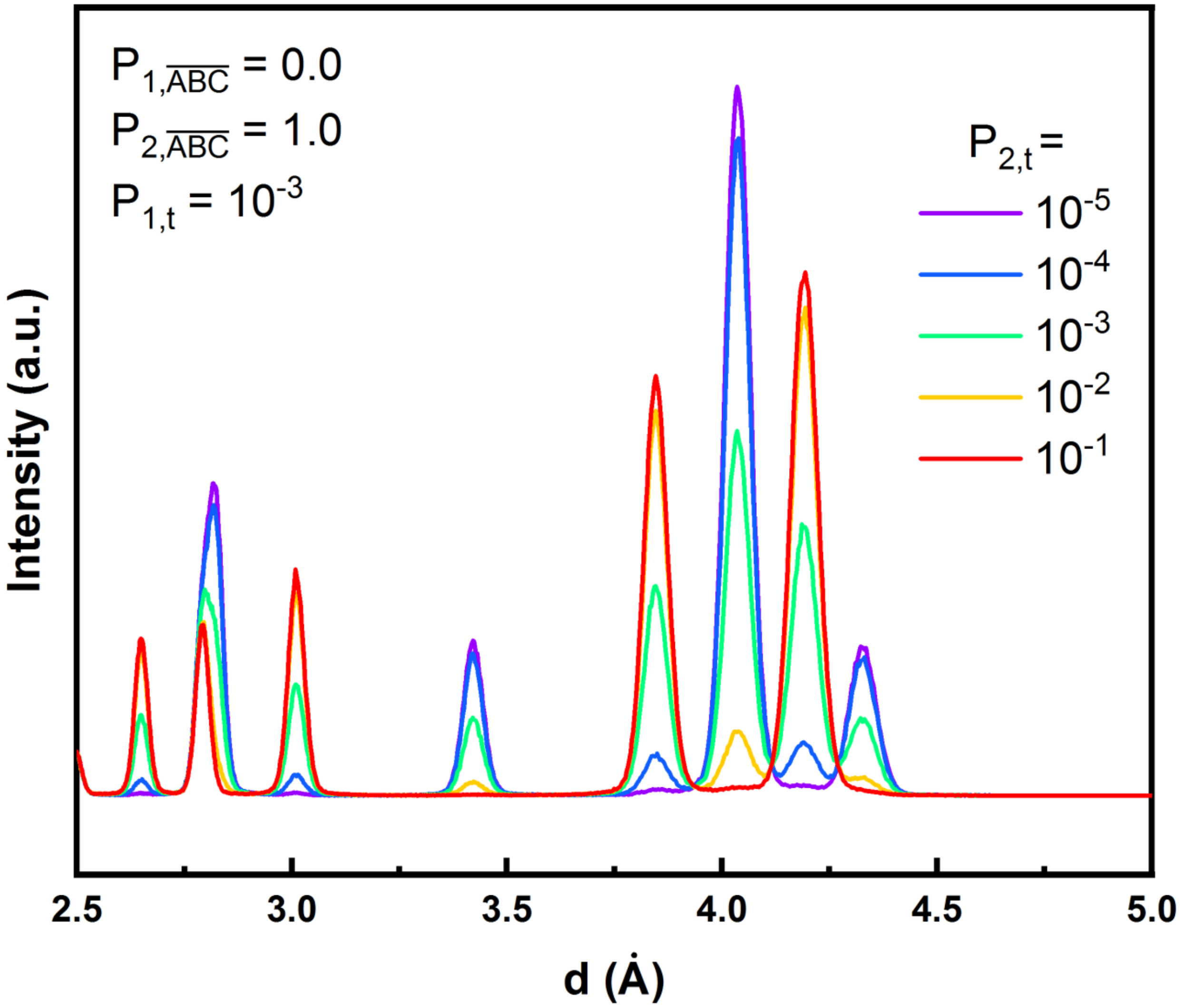

Figure S3. The relative values of $P_{i,t}$ control the layer fraction of the phases. At $P_{2,t}/P_{1,t} = 0.01$ (purple), the ABAB ordered phase dominates. At $P_{2,t}/P_{1,t} = 100$ (red), the ABCABC ordered phase dominates. At $P_{2,t}/P_{1,t} = 1$, the ABAB and ABCABC domains are present in equal amounts.

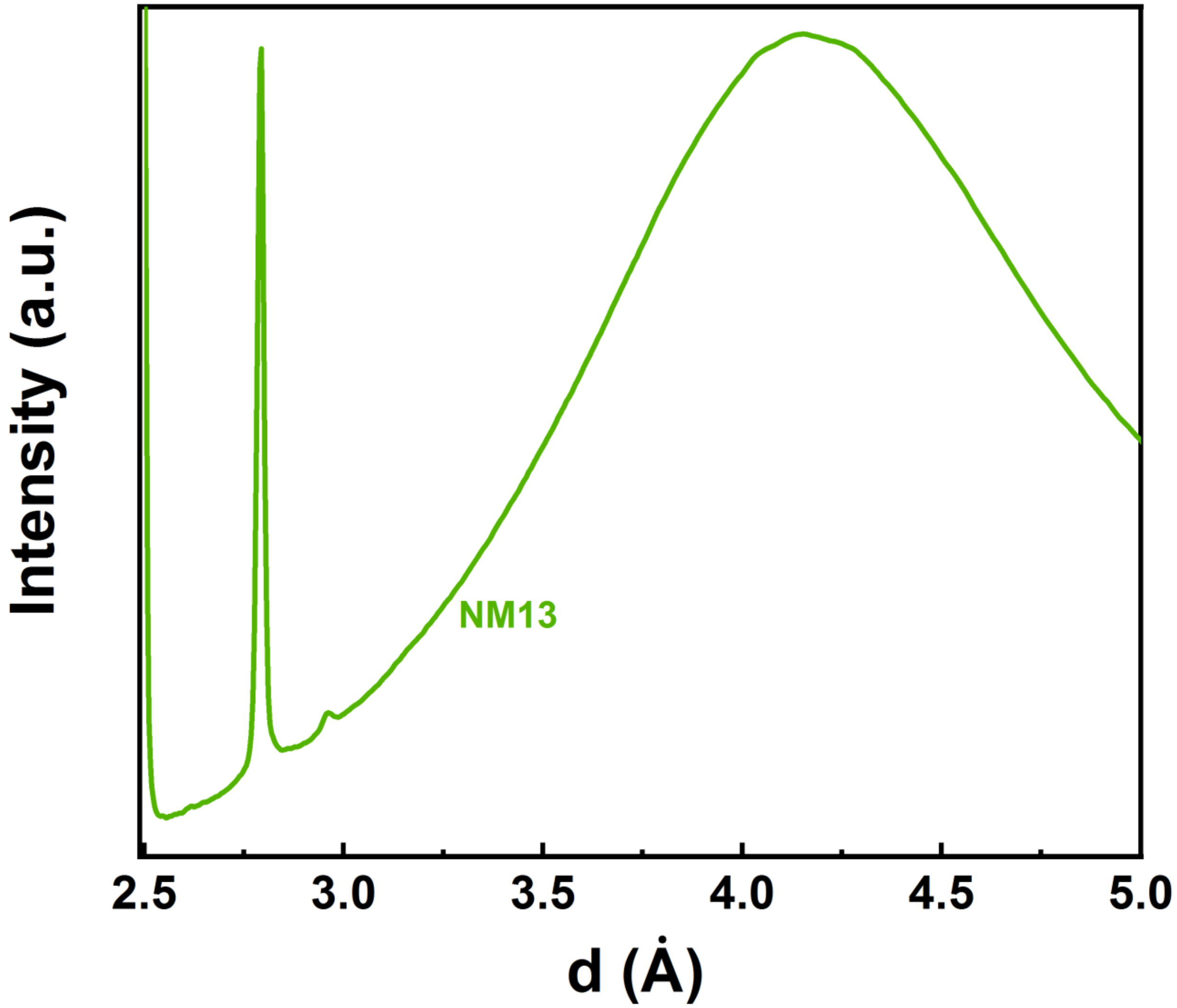

Figure S4. The XRD pattern of NM13 without background subtraction.

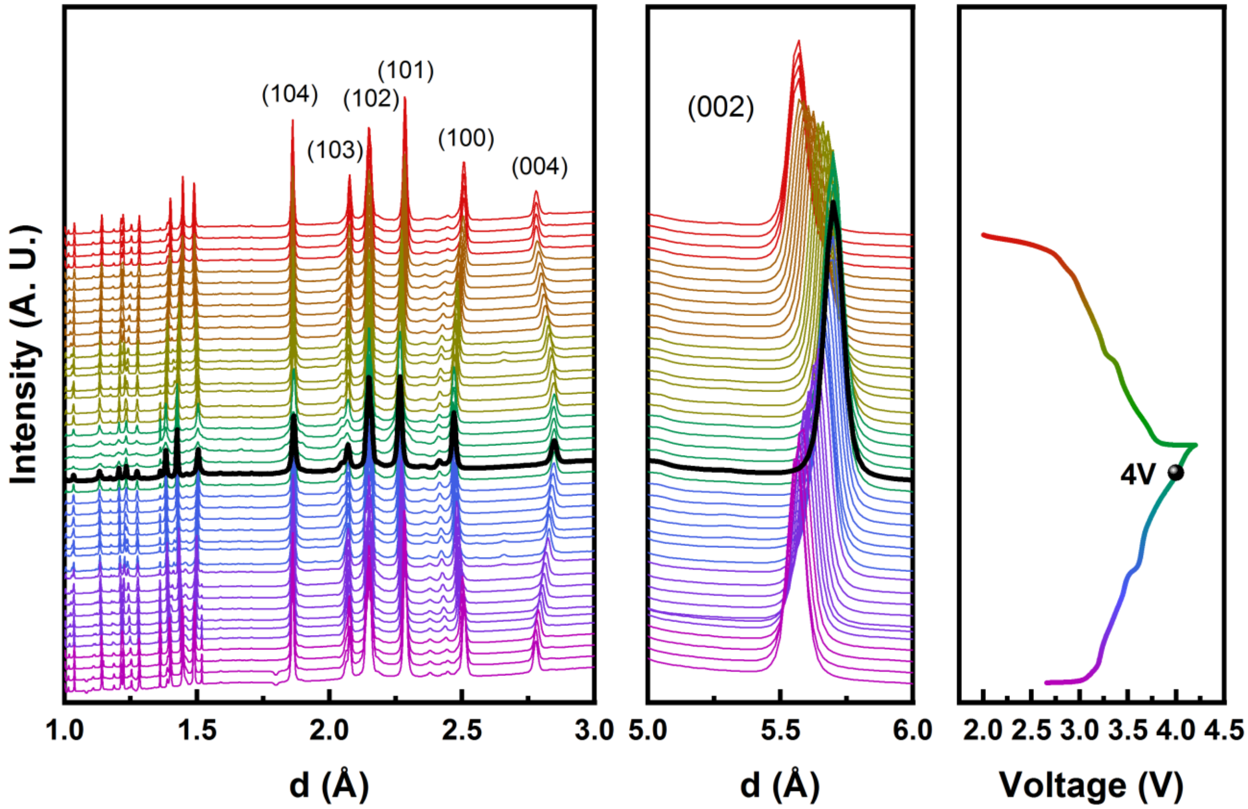

Figure S5. Operando sXRD of LFN10 during charge/discharge between 2-4.2V. Peaks are indexed to the typical P6$_3$/mmc unit cell (a x a x c).

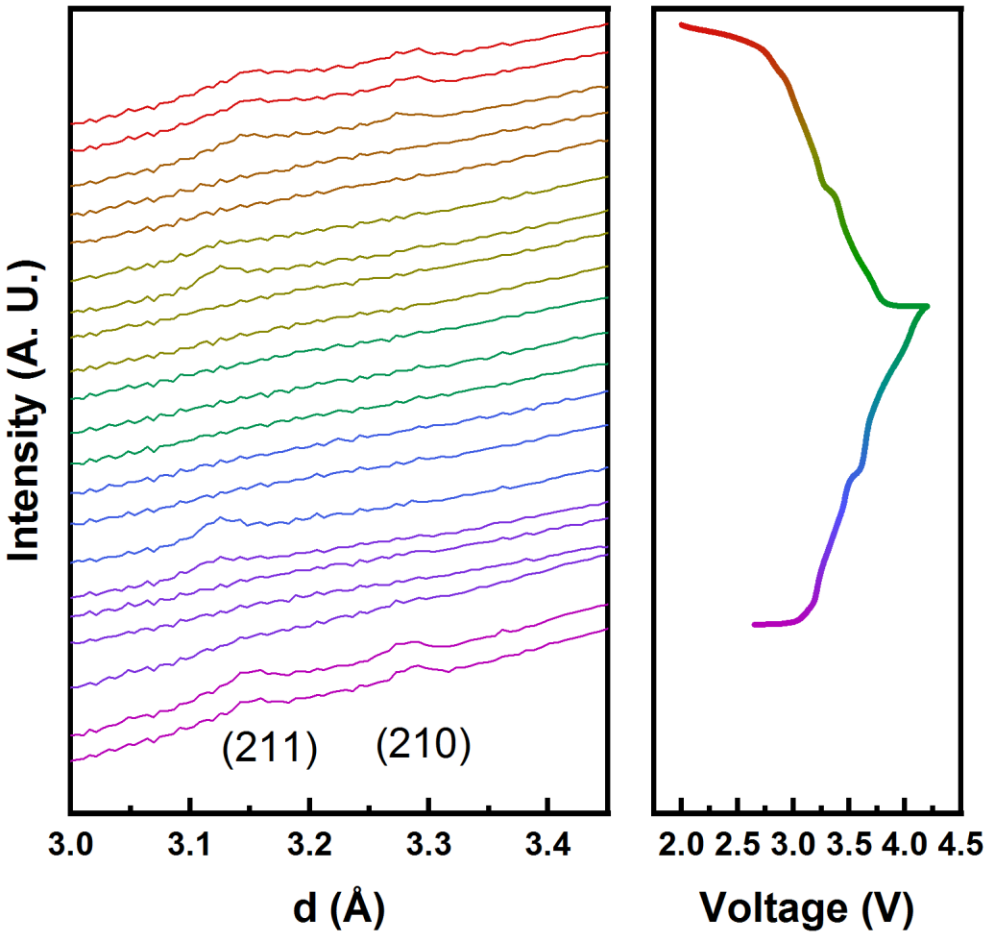

Figure S6. Zoomed in view of operando sXRD pattern of LFN10 highlighting the Na^+/vacancy ordering peaks during charge/discharge between 2-4.2V. Peaks are indexed according to the $2\sqrt{3}a \times 2\sqrt{3}a \times c$ unit cell that captures the LZZ Na^+/vacancy ordering.

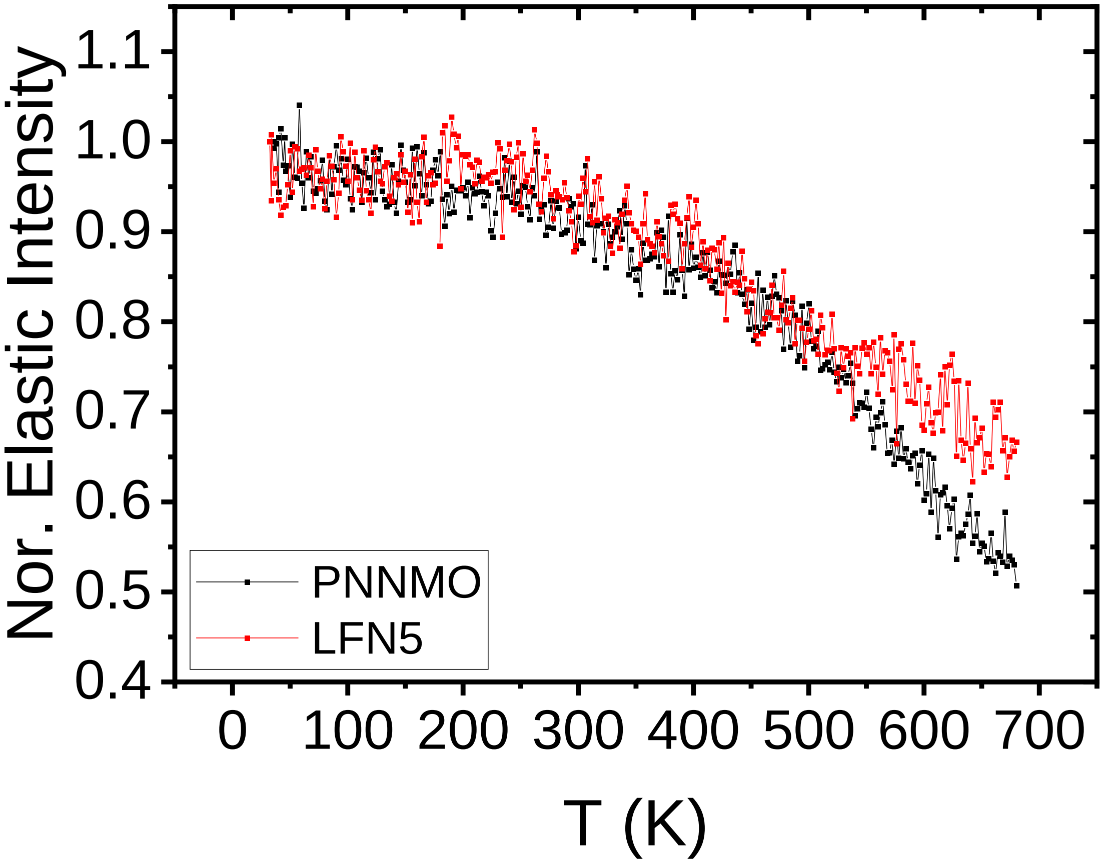

Figure S7. Normalized elastic intensity scan of PNNMO and LFN5 samples as a function of temperatures.

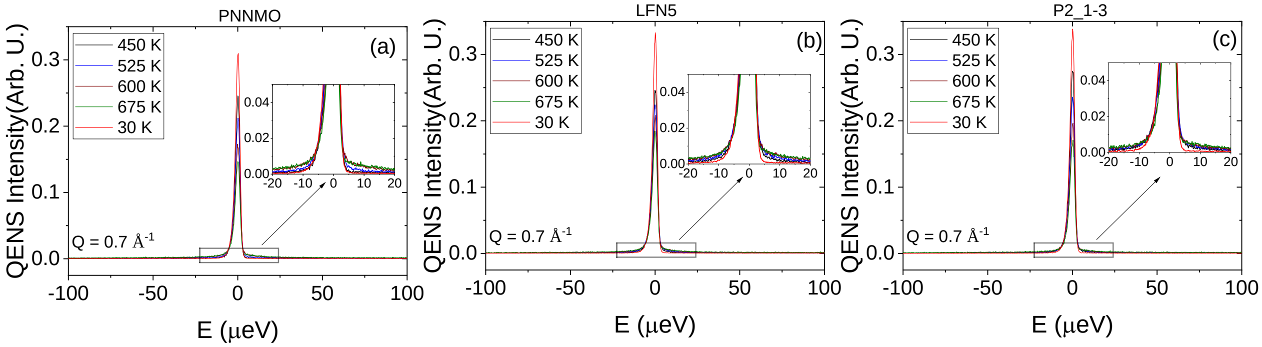

Figure S8. Experimental QENS signals at different temperatures at a representative Q = 0.7 $Å^{-1}$ (a) PNNMO (b) LFN5, and (c) NM13 (P2_1-3). Insets in all three figures are zoomed regions pointed by arrows to visualize the quasielastic broadening.

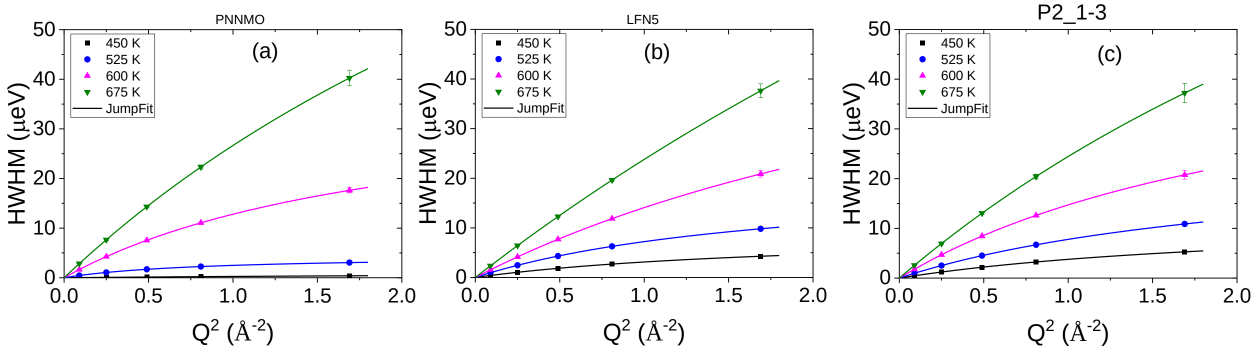

Figure S9. Q-dependence of HWHM of the QENS spectra at indicated temperatures (a) PNNMO (b) LFN5, and (c) NM13 (P2_1-3). Solid lines are the fit from the jump-diffusion model.

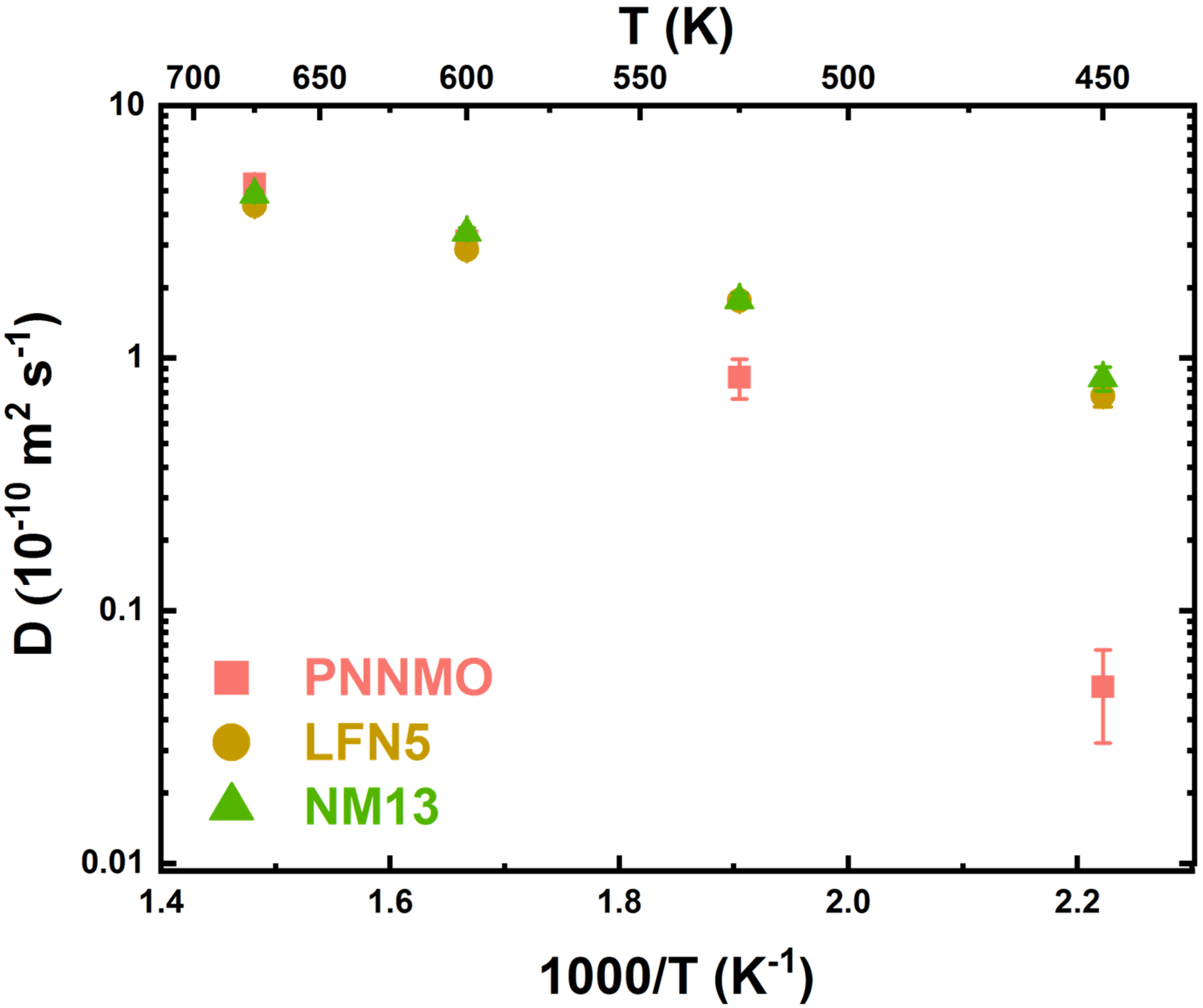

Figure S10. Diffusivity obtained from QENS measurement as a function of (inverse) temperature.

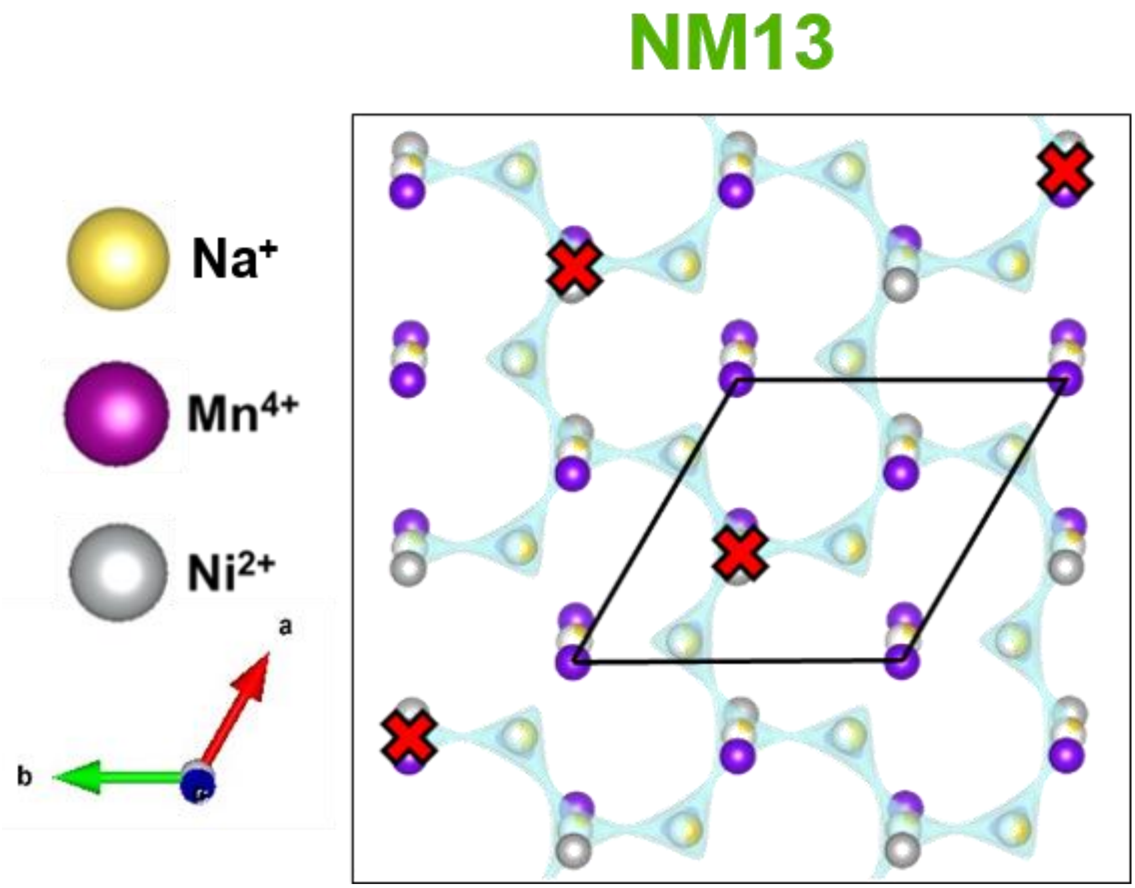

Figure S11. Schematic of the disruption caused by the higher Mn content in NM13 compared to PNNMO, where the red crosses indicate Ni-Mn sites that would be replaced by Mn-Mn sites. The blue isosurfaces are those calculated for PNNMO, which would be blocked at the sites indicated by the crosses.

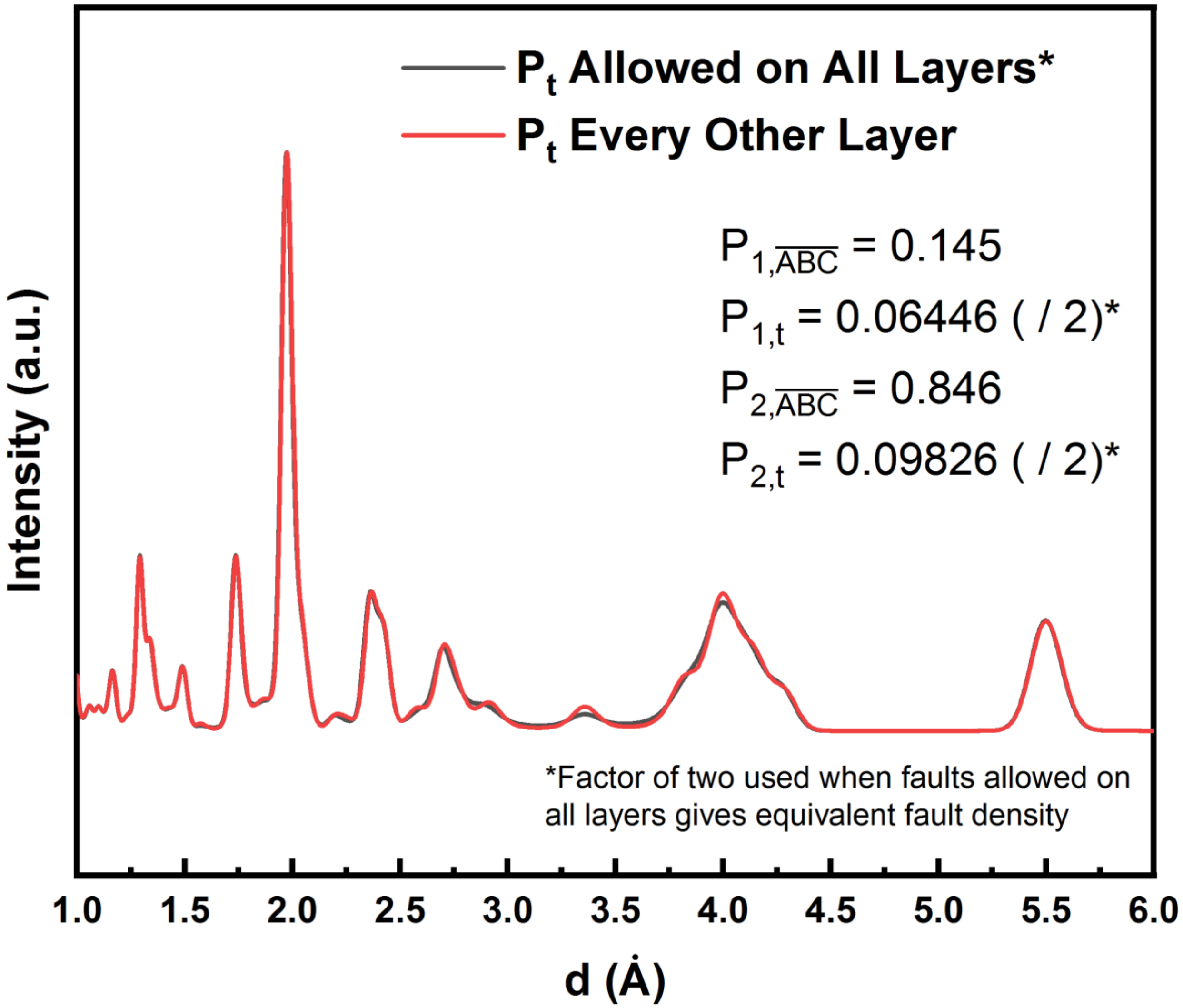

Figure S12. Comparison of the calculated diffraction patterns with equivalent parameters other than whether faults may occur on all layers (as in simulations) or every other layer (necessary for refinement).

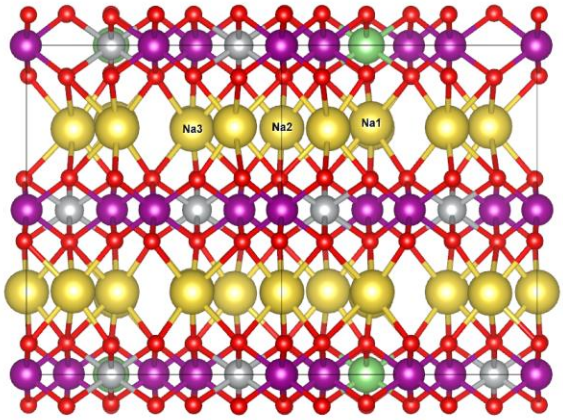

Figure S13. Optimized crystal structures for calculating site energies with a $Na_{16}Li_1Mn_{16}Ni_7O_{48}$ structure. The Na1, Na2, and Na3 atoms are at Mn-Li, Mn-Mn, and Mn-Ni coordination.

Table S1. ICP-MS results for the materials (normalizing the sum of the transition metals to 1.00):

Sample	Na	Li	Ni	Mn
PNNMO	0.6874	0.0001	0.3346	0.6654
NM13	0.6653	0.0004	0.2636	0.7364
LSN10	0.5992	0.1074	0.3353	0.6647
LFN10	0.6978	0.1083	0.3369	0.6631

Table S2. Refinement results of faulting model for PNNMO, NM13, and LFN5.

**Layer Structure**					
**PNNMO & NM13: Layer 1 (= 2 = 3)**					
**LFN5: Layer 1 (= 2 = 3 = 4 = 5 = 6)**					
**Atom**	**x**	**y**	**z**	**B_{iso}**	**Occupancy**
**Ni^{2+}**	0	0	0	$B_{iso,TM}$	Ni_A-Mn_{Ni}
**Mn^{4+}**	1/3	2/3	0	$B_{iso,TM}$	1-(Mn_{Ni}/2)
**Mn^{4+}**	2/3	1/3	0	$B_{iso,TM}$	1-(Mn_{Ni}/2)
**Mn^{4+}**	0	0	0	$B_{iso,TM}$	1-Ni_A+Mn_{Ni}
**Ni^{2+}**	1/3	2/3	0	$B_{iso,TM}$	Mn_{Ni}/2
**Ni^{2+}**	2/3	1/3	0	$B_{iso,TM}$	Mn_{Ni}/2
**Na^{1+}**	0	0	0.25	$B_{iso,Na}$	0.67 - $Occ_{Na,E}$
**Na^{1+}**	1/3	2/3	0.25	$B_{iso,Na}$	0.67 - $Occ_{Na,E}$
**Na^{1+}**	2/3	1/3	0.25	$B_{iso,Na}$	0.67 - $Occ_{Na,E}$
**Na^{1+}**	0	1/3	0.25	$B_{iso,Na}$	$Occ_{Na,E}$
**Na^{1+}**	2/3	2/3	0.25	$B_{iso,Na}$	$Occ_{Na,E}$
**Na^{1+}**	1/3	0	0.25	$B_{iso,Na}$	$Occ_{Na,E}$
**O^{2-}**	1/3 + δ_O	1/3 + δ_O	z_O	$B_{iso,O}$	1
**O^{2-}**	2/3 - δ_O	2/3 - δ_O	- z_O	$B_{iso,O}$	1
**O^{2-}**	2/3-δ	0	z_O	$B_{iso,O}$	1
**O^{2-}**	0	1/3 + δ_O	- z_O	$B_{iso,O}$	1
**O^{2-}**	0	2/3 - δ_O	z_O	$B_{iso,O}$	1
**O^{2-}**	1/3 + δ_O	0	- z_O	$B_{iso,O}$	1

**PNNMO & NM13: Layer 4 (= 5 = 6)**					
**LFN5: Layer 7 (= 8 = 9 = 10 = 11 = 12)**					
**Atom**	**x**	**y**	**z**	**Biso**	**Occupancy**
**Ni^{2+}**	0	0	0	$B_{iso,TM}$	Ni_A-Mn_{Ni}
**Mn^{4+}**	1/3	2/3	0	$B_{iso,TM}$	1-(Mn_{Ni}/2)
**Mn^{4+}**	2/3	1/3	0	$B_{iso,TM}$	1-(Mn_{Ni}/2)
**Mn^{4+}**	0	0	0	$B_{iso,TM}$	1-Ni_A+Mn_{Ni}
**Ni^{2+}**	1/3	2/3	0	$B_{iso,TM}$	Mn_{Ni}/2
**Ni^{2+}**	2/3	1/3	0	$B_{iso,TM}$	Mn_{Ni}/2
**Na^{1+}**	0	0	0.25	$B_{iso,Na}$	0.67 - $Occ_{Na,E}$
**Na^{1+}**	1/3	2/3	0.25	$B_{iso,Na}$	0.67 - $Occ_{Na,E}$
**Na^{1+}**	2/3	1/3	0.25	$B_{iso,Na}$	0.67 - $Occ_{Na,E}$
**Na^{1+}**	1/3	1/3	0.25	$B_{iso,Na}$	$Occ_{Na,E}$
**Na^{1+}**	0	2/3	0.25	$B_{iso,Na}$	$Occ_{Na,E}$
**Na^{1+}**	2/3	0	0.25	$B_{iso,Na}$	$Occ_{Na,E}$
**O^{2-}**	1/3 + δ_O	1/3 + δ_O	- z_O	$B_{iso,O}$	1
**O^{2-}**	2/3 - δ_O	2/3 - δ_O	z_O	$B_{iso,O}$	1
**O^{2-}**	2/3 - δ	0	- z_O	$B_{iso,O}$	1
**O^{2-}**	0	1/3 + δ_O	z_O	$B_{iso,O}$	1
**O^{2-}**	0	2/3 - δ_O	- z_O	$B_{iso,O}$	1

**O^{2-}**	$1/3 + \delta_O$	0	z_O	$B_{iso,O}$	1

Transitions for PNNMO & NM13							
**Layering Scheme**	**i**	**j**	**Probability (α_{ij})**	**Vector Label**	**$R_{x,ij}$**	**$R_{y,ij}$**	**$R_{z,ij}$**
**AB-Rich**	1	5	$1-P_{ABC}$	R_{AB}	2/3	1/3	1/2
	1	6	P_{ABC}	R_{ABC}	1/3	2/3	1/2
	2	4	P_{ABC}	R_{ABC}	1/3	2/3	1/2
	2	6	$1-P_{ABC}$	R_{AB}	2/3	1/3	1/2
	3	4	$1-P_{ABC}$	R_{AB}	2/3	1/3	1/2
	3	5	P_{ABC}	R_{ABC}	1/3	2/3	1/2
	4	2	P_{ABC}	R_{AB}	2/3	1/3	1/2
	4	3	$1-P_{ABC}$	R_{ABC}	1/3	2/3	1/2
	5	1	$1-P_{ABC}$	R_{ABC}	1/3	2/3	1/2
	5	3	P_{ABC}	R_{AB}	2/3	1/3	1/2
	6	1	P_{ABC}	R_{AB}	2/3	1/3	1/2
	6	2	$1-P_{ABC}$	R_{ABC}	1/3	2/3	1/2

Transitions for LFN5							
**Layering Scheme**	**i**	**j**	**Probability (α_{ij})**	**Vector Label**	**$R_{x,ij}$**	**$R_{y,ij}$**	**$R_{z,ij}$**
AB-Rich	1	8	$1-P_{1,t}$	$R_{1,ABC}$	2/3	1/3	1/2
	1	11	$P_{1,t}$	$R_{1,t}$	2/3	1/3	1/2
	2	9	$1- P_{1,t}$	$R_{1,ABC}$	2/3	1/3	1/2
	2	12	$P_{1,t}$	$R_{1,t}$	2/3	1/3	1/2
	3	7	$1- P_{1,t}$	$R_{1,ABC}$	2/3	1/3	1/2
	3	10	$P_{1,t}$	$R_{1,t}$	2/3	1/3	1/2
ABC-Rich	4	8	$P_{2,t}$	$R_{2,ABC}$	2/3	1/3	$1/2+ \delta_{ABC}$
	4	11	$1- P_{2,t}$	$R_{2,t}$	2/3	1/3	$1/2+ \delta_{ABC}$
	5	9	$P_{2,t}$	$R_{2,ABC}$	2/3	1/3	$1/2+ \delta_{ABC}$
	5	12	$1- P_{2,t}$	$R_{2,t}$	2/3	1/3	$1/2+ \delta_{ABC}$
	6	7	$P_{2,t}$	$R_{2,ABC}$	2/3	1/3	$1/2+ \delta_{ABC}$
	6	10	$1- P_{2,t}$	$R_{2,t}$	2/3	1/3	$1/2+ \delta_{ABC}$
AB-Rich	7	2	$P_{1,ABC}$	$R_{1,ABC}$	2/3	1/3	1/2
	7	3	$P_{1,AB}$	$R_{1,AB}$	1/3	2/3	1/2
	8	1	$P_{1,AB}$	$R_{1,AB}$	1/3	2/3	1/2
	8	3	$P_{1,ABC}$	$R_{1,ABC}$	2/3	1/3	1/2
	9	1	$P_{1,ABC}$	$R_{1,ABC}$	2/3	1/3	1/2
	9	2	$P_{1,AB}$	$R_{1,AB}$	1/3	2/3	1/2
ABC-Rich	10	5	$P_{2,ABC}$	$R_{2,ABC}$	2/3	1/3	$1/2+ \delta_{ABC}$
	10	6	$P_{2,AB}$	$R_{2,AB}$	1/3	2/3	$1/2+ \delta_{ABC}$
	11	4	$P_{2,AB}$	$R_{2,AB}$	1/3	2/3	$1/2+ \delta_{ABC}$
	11	6	$P_{2,ABC}$	$R_{2,ABC}$	2/3	1/3	$1/2+ \delta_{ABC}$
	12	4	$P_{2,ABC}$	$R_{2,ABC}$	2/3	1/3	1/2
	12	5	$P_{2,AB}$	$R_{2,AB}$	1/3	2/3	1/2

Refined Parameters, Defined Constants, and Fit Statistics							
	**PNNMO**		**NM13**		**LFN5**		**Units**
**Parameter**	**Final Value**	**Standard Deviation**	**Final Value**	**Standard Deviation**	**Final Value**	**Standard Deviation**	
R_{wp}	6.48	-	2.22	-	6.15	-	%
Ni_A	1	-	0.75	-	1	-	-
a	5.00630	0.00292	5.00608	0.00014	5.00835	0.00028	Å
c	11.13217	0.00056	11.15079	0.00272	11.05749	0.00240	Å
$B_{iso,TM}$	0.63863	0.11473	1.80561	0.20159	1.30458	0.12032	$Å^2$
$B_{iso,Na}$	1.83900	0.20412	0.51759	0.16934	0.06911	0.13477	$Å^2$
$B_{iso,O}$	1.71587	0.08314	1.61061	0.07546	1.37024	0.06907	$Å^2$
δ_O	0.02081	0.00048	0.023857	0.00048	0.017766	0.00044	-
z_O	0.09107	0.00015	0.09156	0.00015	0.09474	0.00013	-
$Occ_{Na,E}$	0.38303	0.00514	0.42744	0.00441	0.43649	0.00406	-
Mn_{Ni}	0.09629	0.00468	0.25765	0.00470	0.09146	0.00426	-
$P_{1,ABC}$	0.27804	0.00509	0.06751	0.00302	0.145339	0.03025	-
$P_{2,ABC}$	-	-	-	-	0.845700	0.03265	-
$P_{1,t}$	-	-	-	-	0.064460	0.04450	-
$P_{2,t}$	-	-	-	-	0.098259	0.06415	-
δ_{ABC}	-	-	-	-	0.00067	0.00036	-

Table S3. Rietveld refinement results of PNNMO, NM13, and LFN5.

ABAB Ni/Mn Ordered P2 – Space group: C2					
**a' = √3a, b = 3a, c'=c, β=90.0°**					
**Atom**	**x**	**y**	**z**	**Occupancy**	**Wyckoff**
**Ni^{2+}**	0	0	0	Ni_A-Mn_{Ni}	2a
**Ni^{2+}**	1/2	1/6	1/2	Ni_A-Mn_{Ni}	2a
**Mn^{4+}**	1/2	1/6	0	1-(Mn_{Ni}/2)	2a
**Mn^{4+}**	0	1/3	0	1-(Mn_{Ni}/2)	2a
**Mn^{4+}**	0	0	1/2	1-(Mn_{Ni}/2)	2b
**Mn^{4+}**	0	1/3	1/2	1-(Mn_{Ni}/2)	2b
**Mn^{4+}**	0	0	0	1-Ni_A+Mn_{Ni}	2a
**Mn^{4+}**	1/2	1/6	1/2	1-Ni_A+Mn_{Ni}	2a
**Ni^{2+}**	1/2	1/6	0	Mn_{Ni}/2	2a
**Ni^{2+}**	0	1/3	0	Mn_{Ni}/2	2a
**Ni^{2+}**	0	0	1/2	Mn_{Ni}/2	2b
**Ni^{2+}**	0	1/3	1/2	Mn_{Ni}/2	2b
**O^{2-}**	1/3 + δO	0	z_O	1	4c
**O^{2-}**	-1/6 - (δO/2)	1/6 + (δO/2)	z_O	1	4c
**O^{2-}**	-1/6 - (δO/2)	-1/6 - (δO/2)	z_O	1	4c
**O^{2-}**	1/3 - (δO/2)	-(δO/2)	1/2 - z_O	1	4c
**O^{2-}**	-1/6 + δO	1/6	1/2 - z_O	1	4c
**O^{2-}**	1/3 - (δO/2)	1/3 + (δO/2)	1/2 - z_O	1	4c
**Na^{+}**	0	0	1/4	2/3 – $Occ_{Na,E}$	4c
**Na^{+}**	0	1/3	1/4	2/3 – $Occ_{Na,E}$	4c
**Na^{+}**	0	2/3	1/4	2/3 – $Occ_{Na,E}$	4c
**Na^{+}**	1/6	1/6	1/4	$Occ_{Na,E}$	4c
**Na^{+}**	2/3	0	1/4	$Occ_{Na,E}$	4c
**Na^{+}**	2/3	1/3	1/4	$Occ_{Na,E}$	4c

ABCABC Ni/Mn Ordered P2 – Space group: R-3c					
**a' = √3a, c'=3c, β=90.0°**					
**Atom**	**x**	**y**	**z**	**Occupancy**	**Wyckoff**
**Ni^{2+}**	0	0	0	Ni_A-Mn_{Ni}	6b
**Mn^{4+}**	0	0	1/6	1-(Mn_{Ni}/2)	12c
**Mn^{4+}**	0	0	0	Mn_{Ni}	6b
**Ni^{2+}**	0	0	1/6	Mn_{Ni}/2	12c
**O^{2-}**	1/3 + δ_O	0	z_O/3	1	36f
**Na^+**	0	0	1/4	2/3 – $Occ_{Na,E}$	6a
**Na^+**	1/3	2/3	1/4	2/3 – $Occ_{Na,E}$	12c
**Na^+**	2/3	2/3	1/4	$Occ_{Na,E}$	18e

	**Refined Parameters, Defined Constants, and Fit Statistics**							
		**PNNMO**		**NM13**		**LFN5**		
	**Parameter**	**Final Value**	**Std. Dev.**	**Final Value**	**Std. Dev.**	**Final Value**	**Std. Dev.**	**Units**
	R_{wp}	10.185	-	2.59	-	7.46	-	%
	Ni_A	1	-	3/4	-	1	-	-
ABAB (C2)	*a*	5.01565	0.00020	5.01460	0.00006	5.01558	0.00112	-
	b (=√3a)	8.68736	0.00034	8.68555	0.00010	8.68724	0.00193	Å
	c	11.17291	0.00392	11.16000	0.00107	11.08294	0.00620	Å
	β	90	-	90	-	90.00000	-	°
	wt%	100	-	100	-	59.83400	0.93700	wt%
ABCABC (R-3c)	*a*	-	-	-	-	5.01087	0.00325	Å
	c	-	-	-	-	33.34558	0.02376	Å
	wt%	0	-	0	-	40.16600	0.93700	wt%
Shared Parameters	$U_{iso,TM}$	0.01007	0.00364	0.00251	0.00056	0.00119	0.00952	$Å^2$
	$U_{iso,Na}$	0.01421	0.00506	0.04086	0.00143	0.06866	0.01994	$Å^2$
	$U_{iso,O}$	0.02635	0.00220	0.00952	0.00035	0.01540	0.00483	$Å^2$
	δ_O	0.01135	0.00038	0.02068	0.00009	0.01666	0.00033	-
	z_O	0.09577	0.00190	0.09208	0.01360	0.09070	0.00026	-
	$Occ_{Na,E}$	0.41460	0.01100	0.40900	0.00143	0.46800	0.00900	-
	Mn_{Ni}	0.19780	0.01200	0.29800	0.00200	0.13000	0.00500	-
	D_G	271.4	10	230.4	3	450.4	16	Å

Experimental Methods:

Synthesis:

P2 $Na_{0.67}Ni_{0.33}Mn_{0.67}O_2$ was prepared by a solid-state reaction using the $Ni_{0.33}Mn_{0.67}(OH)_2$ precursor with Na_2CO_3. The $Ni_{0.33}Mn_{0.67}(OH)_2$ precursor was prepared by a co-precipitation method adopted from the work by Dahn et al.[1] A 1.0M total solution of $NiSO_4$ and $MnSO_4$ was prepared from $NiSO_4$-$6H_2O$ (99%, Acros) and $MnSO_4$-H_2O (99%+, Thermo Scientific) salts were pumped at a constant rate of 0.1g/min into a 250mL vessel initially charged with 125mL of 0.37M NH_3-H_2O (Fisher) solution. The reaction was held at a constant 60°C and kept under an argon atmosphere to prevent oxidation of the precipitate. pH was kept constant at 9.8±0.1 regulating the flow of 1M NaOH (98%, Fisher) solution with a valve operated by a pH controller (Cole Parmer pH/ORP 400). All solutions were prepared with ultrapure water (18.2 MOhm-cm). The volume in the reactor was held constant by pumping excess volume out of the reactor. The reactor was continuously stirred at a constant rate by a magnetic stir bar. The reaction was allowed to proceed for at least 3 hours before collecting the precipitated product in an argon-purged filter funnel. The collected precipitate was thoroughly rinsed with 3L ultrapure water that was deoxygenated by boiling and then by actively bubbling argon for at least 5 minutes. The rinsed precipitate was then dried under vacuum at 70°C overnight before being transferred into an argon-filled glovebox for storage to prevent oxidation. The powder was lightly ground to pass through a 150um sieve prior to further use. The stoichiometric ratio of nickel to manganese was verified to be the designed 1:2 by energy dispersive x-ray spectroscopy. $Ni_{0.25}Mn_{0.75}(OH)_2$ was prepared by an identical method with appropriate modification to the quantity of the Ni and Mn sulfates.

The $TM(OH)_2$ powder and a proportional amount of Na_2CO_3 (99.95% anhydrous, Acros) and Li_2CO_3 (99%, Sigma Aldrich) were combined in stoichiometric amounts totaling 1 gram and were thoroughly mixed 3 times for 2 minutes at 2000RPM using a planetary mixer (THINKY) with zirconia balls with manual intermediate stirring. A 5% molar excess of sodium was used to compensate for evaporation at high temperatures. Samples prepared for the NPD and QENS measurements used ^7Li enriched Li_2CO_3 (99%, Sigma Aldrich) to minimize the high neutron absorption of ^6Li. The mixed powders were formed into a 12.7mm diameter pellet with a hydraulic press (Carver) at 5 metric tons. The pelletized material was calcined in alumina crucibles by heating at 5°C/min to 850 °C and held for 20 h in the air using a muffle furnace (Neytech Vulcan 3-550A). The furnace and samples cooled naturally to 500 °C before quenching in air. The quenched pellets were transferred to the glovebox to avoid exposure to the moisture in the air. The pellets were lightly ground until they could pass through a 400 mesh (38um) sieve prior to electrode preparation.

Electrochemical Characterization:

All electrochemical testing was conducted with 1M $NaPF_6$ in propylene carbonate as the electrolyte with glassy fiber separators against sodium metal. Electrodes were prepared from 80 wt% active material, 10 wt% carbon (C45, Timcal), and 10 wt% PVDF (from 8wt% PVDF in n-methyl pyrrolidone) that were dried overnight at 70°C. Typical active material loading was 2-3 mg/cm^2. Galvanostatic cycling (for voltage profiles and rate study) was conducted in CR2032-type coinc cells using an Arbin battery cycler. Operando sXRD was conducted in coin cells with a Kapton window to allow x-ray transmission using a Maccor battery cycler.

Physical Characterization:

sXRD: The powders were loaded into 1mm Kapton capillaries and sealed with modeling clay immediately prior to measurement. Diffraction measurements were conducted at sector 11-ID-C of the Advanced Photon Source at Argonne National Laboratory (samples PNNMO, LFN5, LFN10, LFN20, LSN10) with x-ray wavelength of 0.1173 Å, and at sector 28-ID-2 of the National Synchrotron Light Source II (NSLS-II) at Brookhaven National Laboratory (sample NM13) with a x-ray wavelength of 0.1814 Å. The data collected at 11-ID-C are reproduced from our previous publication.[2] Operando sXRD was conducted at beamline 17-BM of the Advanced Photon Source with a wavelength of 0.24145 Å.

NPD: The powders (~0.2g) were loaded into 3mm quartz capillaries and sealed with epoxy inside an argon-filled glovebox (<0.5ppm O_2) prior to measurement at the Nanoscale-Ordered Materials Diffractometer (NOMAD, BL-1B) instrument at the Spallation Neutron Source facility at Oak Ridge National Laboratory.[3] Two 24 min scans were collected on each powder sample and then summed to improve the counting statistic. Scattering signal from the empty vanadium can measurements was subtracted as background from the sample measurement and data were normalized by the scattering intensity from a 6 mm vanadium rod to correct for detector efficiencies.

ICP-MS: ICP-MS was conducted at the Boise State University Isotope Geology Laboratory to determine the overall composition. The samples were dissolved in 2.5 mL 16M HNO_3 + 2.5mL H_2O and 100 µL 30% H_2O_2, diluted gravimetrically in 2% HNO_3 for analysis. The solution was analyzed by a ThermoScientific, iCAP-RQ, inductively coupled plasma mass spectrometer.

QENS: Quasielastic neutron scattering measurements were conducted at the Backscattering Silicon Spectrometer (BASIS, BL-2) instrument at the Spallation Neutron Source of Oak Ridge National Laboratory.[4] Samples were heated in an argon glovebox to at least 200°C before transfer into the sample canister to remove any adsorbed water. The samples were sealed in an annular aluminum can with a 2 mm gap for the measurement. In this experiment, BASIS was operated at 60 Hz chopper frequency with incident neutron of bandwidth centered at 6. 4 Å. This instrument configuration provides a Q range of 0.2 $Å^{-1}$ to 2.0 $Å^{-1}$ with an energy transfer range of ±100 µeV and an energy resolution of 3.6 µev. Short elastic scattering intensity measurements as a function of temperature was performed from 30 K to 680 K at a 1K/min heating rate. Long QENS spectra were collected from each sample at 450 K, 525 K, 600 K, and 675 K. The temperatures were maintained using closed cycle refrigerator with helium exchange gas. Sample specific instrument resolution function was measured at 30 K. Data from an empty can at 675 K were collected and subtracted as a background. Standard vanadium dada was used for detector efficiency normalization. QENS data were reduced in the Mantid package[5] and analyzed using QClimax software.[6] The QENS intensity signal, *I (Q,E)*, were fitted with a one component model as:

$$I(Q,E) = \left[X_1(Q)\delta(E) + \left(1 - X_1(Q)\right)S(Q,E) + B(Q,E)\right] \otimes R(Q,E) \quad (1)$$

where $X_1(Q)$ captures the elastic scattering fraction. The delta function, $\delta(E)$, which is centered at zero energy transfer, accounts the elastic contribution. The dynamic structure factor, $S(Q,E)$, contains the information about the mobility of Na ions in the samples. A data at each Q were fitted after adding a linear background, *(B(Q,E))* and a convolution ($\otimes$) with the instrument resolution

function, $R(Q,E)$. A single Lorentzian function was used to model $S(Q,E)$. Therefore, $S(Q,E)$ can be written as:

$$S(Q,E) = \frac{1}{\pi}\frac{\Gamma(Q)}{\Gamma^2(Q)+E^2} \qquad (2)$$

In equation 2, Γ is the half width at half maximum (HWHM) of quasi elastic signal. Q- dependence of Γ was further analyzed using a jump diffusion model, $\Gamma(Q) = \frac{DQ^2}{1+DQ^2\tau_o}$, from which the diffusion coefficient (D) and the residence time (τ_o) are obtained. These parameters can be used to obtain a jump length *(L)* as, $L^2 = 6D\tau_0$.

Simulation and Refinement of the NPD Patterns:

The refinement of the (clustered) faulting model was conducted in FAULTS (based on DiFFAX)[7,8], and traditional Rietveld refinement was performed with GSAS-II.[9] In both cases, the isotropic displacement factors ($B_{iso} = 8\pi^2U_{iso}$), the oxygen positions, and Na site occupancies were constrained to be equivalent between each phase/clustered domain with appropriate modifications made for the different unit cells. The total Na site occupancy was constrained to require the sum of all site occupancies correspond to 0.67Na per formula unit, and the Ni/Mn site occupancy was constrained to match the designed ratio by adjusting the occupancy of Ni on the A site (Ni_A) with an appropriate amount of Mn. Due to the similar scattering behavior of Li and Mn for neutrons ($b_{coh,Mn}$ = -3.73 fm, $b_{coh,Li}$ = -1.9), no adjustment was made for LFN5 to reduce correlation between the refined parameters (Mn_{Ni} especially). The oxygen x and y positions were constrained to allow an expansion of the Ni octahedron and equivalent contraction of the Mn octahedra that preserves a single but distinct bond length for each TM species (Ni-O ~ 2.04 Å, Mn-O ~ 1.91 Å). For the ABAB ordered structure, this reduces the TM-ordered structure (space group: $P6_3$) to monoclinic symmetry (space group: C2, a'=√3a, b=3a, c'=c, β=90.0°), while the ABCABC ordered structure maintains its symmetry (space group: $R\overline{3}c$) but with oxygen x-coordinate allowed to deviate from 1/3. Though not used for any refinements, the AAAA structure can maintain the $P6_3$ space group with similar deviation of the oxygen x-coordinates if Ni is located at the origin.

The GSAS-II refinement used the instrument profile parameters provided by the NOMAD (SNS BL1-B) beamline staff from a fit to SRM 640e Si powder. Due to the software limitation of FAULTS to only handle constant wavelength diffraction data, time-of-flight diffraction data cannot be processed directly in FAULTS. The time-of-flight data was first converted to d-spacing according to the instrument parameters difA, difC, and Zero, and time-of-flight (TOF) according to the equation d = [-difC + (difC^2-4difA(Zero-TOF))]/(2difA). The data were then converted from d-spacing to a pseudo-constant wavelength (pCW) form according to Bragg's law by assuming an artificial wavelength (1.54A) near the center of the instrument's wavelength range. The "pseudo-θ" (θ') derived from this process was used as the x-coordinate for the refinement with FAULTS and included the data from d = 1.0 – 6.8Å. The sensitivity of the refined parameters to the peak shape requires consideration of the difference in profile functions between time-of-flight neutron diffraction and constant-wavelength diffraction. The TOF peak profile consists of a Gaussian component convoluted with two back-to-back exponentials, where the full-width at half maximum (FWHM) Gaussian component (σ) is a function of d-spacing according to $\sigma(d) = \sigma_0 + \sigma_1 d^2 + \sigma_0 d^4$. The Gaussian part of the pCW peak profile is approximated by a pseudo-Voigt function[10] with FWHM that depends on θ according to Γ(θ) =

$Utan^2(\theta)+Vtan(\theta)+W$. To approximate the TOF profile function with the $\Gamma(\theta)$ function the values of U, V, and W were fit to an SRM 640e Si standard after conversion from TOF to θ' to approximate the FWHM as a function of θ'. The simulation of the faulted P2 structures was performed using FAULTS with the same values of U, V, and W as the refinements. We note that in the case of the simulated diffraction patterns, the stacking vectors corresponding to cationic stacking faults are allowed to occur from any layer (the AB or BA layer as the labels relate to the AB BA oxygen stacking sequence). This same procedure is used to refine the single-phase faulted structures of PNNMO and NM13. However, in the case of LFN5 where additional probabilities ($P_{i,t}$) are needed to describe the faulted structure, software limitations do not allow the parameters needed for the mathematical construction of faults occurring on all layers to be refined independently. The probability terms for the stacking faults (i.e., $P_{\overline{AB}}$ and $P_{\overline{ABC}}$) must be multiplied by ($1-P_{i,t}$). To the best of our knowledge, there is no means to refine these probabilities independently (only their product is refinable) if they are used on the same layer in FAULTS. Therefore, for LFN5 we limit the occurrence of in-phase stacking faults ($P_{1,AB}$, $P_{1,ABC}$, or $P_{2,AB}$, $P_{2,ABC}$) to only occur from the 'BA' layer (layer 7 and equivalents as in **Table S1**), while the stacking vectors between phases ($P_{1,t}$, $P_{2,t}$) only occur from the 'AB' Layer (Layer 1 and equivalents in **Table S1**). This has the effect that the refined values of $P_{i,t}$ are double what the equivalent values for the simulation would be. Nonetheless, the simulation of faults occurring on all layers using the equivalent halved value (refined $P_{i,t}/2$ = simulated $P_{i,t}$) results in an extremely similar calculated diffraction pattern and makes the independent refinement of the two parameters possible (**Figure S12**).

For the traditional Rietveld refinement, Scherrer broadening[11,12] was applied to account for the finite crystallite size (D_G) but was found to be unnecessary for the refinement of the faulted structures (taking values >1 μm). In the traditional refinement, the ABAB phase was allowed to relax the *a, b (=√3a),* and *c* lattice parameters while β was held constant at 90°.

Computational Methods:

The calculations were obtained using the Vienna Ab initio Simulation Package (VASP).[13,14] The Perdew−Burke−Ernzerhof (PBE)[15] form of a generalized gradient approximation (GGA)[15] was adapted to describe the electron exchange and correlation effects with effective Hubbard U values of 3.9 eV and 6.2 eV for Mn and Ni, respectively.[16] A plane wave basis set with a cutoff energy of 520 eV and a *k*-point density of more than 1200 per atom were used. These parameters are in line with the Material Project settings.[17] DFT-D3 was applied to correct the van der Waals interactions between layers.[18] All structures were fully relaxed until the force and energy were less than 0.02 eV/Å and 10^{-5} eV. Li doped ABAB and ABCABC structures were made by substituting lithium (Li) in place of nickel or manganese, succeeded by the meticulous ordering of initially disordered structures — where sodium (Na) sites exhibited disorder in the pristine material — leveraging the pymatgen package.[19] Subsequent to the ordering phase, geometry optimizations were applied to attain optimized configurations for both 82 ordered ABAB and 90 ordered ABCABC configurations, respectively. The energy values presented are representative of the most stable configurations of Li/Mn and Li/Ni within both ABAB and ABCABC frameworks.

The site energy was computed by removing a single Na from $Na_{16}Li_1Mn_{16}Ni_7O_{48}$ (corresponding to $Na_{2/3}Li_{1/24}Mn_{2/3}Ni_{7/24}O_2$), which is close to the experimentally synthesized

LFN5 composition (Figure S13). Only Na sites that share faces with the TM layer are considered, and the site energy is given as:

$$E(\text{site}) = E(Na_{16}Li_1Mn_{16}Ni_7O_{48}) - E(Na_{15}v_{M1-M2}Li_1Mn_{16}Ni_7O_{48}) - (E(Na_{16}Li_1Mn_{16}Ni_7O_{48}) - E(Na_{15}v_{\text{edge-sharing}}Li_1Mn_{16}Ni_7O_{48}))$$

Where E refers to the energy, v_{M1-M2} denotes a Na^{+} vacancy in a site sharing faces with M1 and M2 octahedras and $v_{\text{edge-sharing}}$ denotes a Na vacancy in a site sharing edges. Here, the lowest site energy of a Na in an edge-sharing site is used as the zero reference so that the site energy is related to the barrier for Na hopping from an edge-sharing site.

References

(1) van Bommel, A.; Dahn, J. R. Analysis of the Growth Mechanism of Coprecipitated Spherical and Dense Nickel, Manganese, and Cobalt-Containing Hydroxides in the Presence of Aqueous Ammonia. *Chem. Mater.* **2009**, *21* (8), 1500–1503. https://doi.org/10.1021/cm803144d.

(2) Xie, Y.; Gabriel, E.; Fan, L.; Hwang, I.; Li, X.; Zhu, H.; Ren, Y.; Sun, C.; Pipkin, J.; Dustin, M.; Li, M.; Chen, Z.; Lee, E.; Xiong, H. Role of Lithium Doping in P2-Na0.67Ni0.33Mn0.67O2 for Sodium-Ion Batteries. *Chem. Mater.* **2021**, *33* (12), 4445–4455. https://doi.org/10.1021/acs.chemmater.1c00569.

(3) Neuefeind, J.; Feygenson, M.; Carruth, J.; Hoffmann, R.; Chipley, K. K. The Nanoscale Ordered MAterials Diffractometer NOMAD at the Spallation Neutron Source SNS. *Nucl. Instrum. Methods Phys. Res. Sect. B Beam Interact. Mater. At.* **2012**, *287*, 68–75. https://doi.org/10.1016/j.nimb.2012.05.037.

(4) Mamontov, E.; Herwig, K. W. A Time-of-Flight Backscattering Spectrometer at the Spallation Neutron Source, BASIS. *Rev. Sci. Instrum.* **2011**, *82* (8), 085109. https://doi.org/10.1063/1.3626214.

(5) Arnold, O.; Bilheux, J. C.; Borreguero, J. M.; Buts, A.; Campbell, S. I.; Chapon, L.; Doucet, M.; Draper, N.; Ferraz Leal, R.; Gigg, M. A.; Lynch, V. E.; Markvardsen, A.; Mikkelson, D. J.; Mikkelson, R. L.; Miller, R.; Palmen, K.; Parker, P.; Passos, G.; Perring, T. G.; Peterson, P. F.; Ren, S.; Reuter, M. A.; Savici, A. T.; Taylor, J. W.; Taylor, R. J.; Tolchenov, R.; Zhou, W.; Zikovsky, J. Mantid—Data Analysis and Visualization Package for Neutron Scattering and μ SR Experiments. *Nucl. Instrum. Methods Phys. Res. Sect. Accel. Spectrometers Detect. Assoc. Equip.* **2014**, *764*, 156–166. https://doi.org/10.1016/j.nima.2014.07.029.

(6) Mamontov, E.; Smith, R. W.; Billings, J. J.; Ramirez-Cuesta, A. J. Simple Analytical Model for Fitting QENS Data from Liquids. *Phys. B Condens. Matter* **2019**, *566*, 50–54. https://doi.org/10.1016/j.physb.2019.01.051.

(7) Casas-Cabanas, M.; Reynaud, M.; Rikarte, J.; Horbach, P.; Rodriguez-Carvajal, J. FAULTS: A Program for Refinement of Structures with Extended Defects. *J. Appl. Crystallogr.* **2016**, *49* (6), 2259–2269.

(8) Treacy, M.; Newsam, J.; Deem, M. A General Recursion Method for Calculating Diffracted Intensities from Crystals Containing Planar Faults. *Proc. R. Soc. Lond. Ser. Math. Phys. Sci.* **1991**, *433* (1889), 499–520.

(9) Toby, B. H.; Von Dreele, R. B. GSAS-II: The Genesis of a Modern Open-Source All Purpose Crystallography Software Package. *J. Appl. Crystallogr.* **2013**, *46* (2), 544–549. https://doi.org/10.1107/S0021889813003531.

(10) Thompson, P.; Cox, D. E.; Hastings, J. B. Rietveld Refinement of Debye–Scherrer Synchrotron X-Ray Data from Al2O3. *J. Appl. Crystallogr.* **1987**, *20* (2), 79–83. https://doi.org/10.1107/S0021889887087090.
(11) Scherrer, P. Nachr Ges Wiss Goettingen. *Math Phys* **1918**, *2*, 98–100.
(12) Holzwarth, U.; Gibson, N. The Scherrer Equation versus the "Debye-Scherrer Equation." *Nat. Nanotechnol.* **2011**, *6* (9), 534–534. https://doi.org/10.1038/nnano.2011.145.
(13) Kresse, G.; Furthmuller, J. Efficient Iterative Schemes for Ab Initio Total-Energy Calculations Using a Plane-Wave Basis Set. *Phys. Rev. B* **1996**, *54* (16), 11169–11186. https://doi.org/10.1103/PhysRevB.54.11169.
(14) Blöchl, P. E. Projector Augmented-Wave Method. *Phys. Rev. B* **1994**, *50* (24), 17953–17979. https://doi.org/10.1103/PhysRevB.50.17953.
(15) Perdew, J. P.; Burke, K.; Ernzerhof, M. Generalized Gradient Approximation Made Simple. *Phys. Rev. Lett.* **1996**, *77* (18), 3865–3868. https://doi.org/10.1103/PhysRevLett.77.3865.
(16) Dudarev, S. L.; Botton, G. A.; Savrasov, S. Y.; Humphreys, C. J.; Sutton, A. P. Electron-Energy-Loss Spectra and the Structural Stability of Nickel Oxide: An LSDA+U Study. *Phys. Rev. B* **1998**, *57* (3), 1505–1509. https://doi.org/10.1103/PhysRevB.57.1505.
(17) Jain, A.; Ong, S. P.; Hautier, G.; Chen, W.; Richards, W. D.; Dacek, S.; Cholia, S.; Gunter, D.; Skinner, D.; Ceder, G.; Persson, K. A. Commentary: The Materials Project: A Materials Genome Approach to Accelerating Materials Innovation. *APL Mater.* **2013**, *1* (1), 011002. https://doi.org/10.1063/1.4812323.
(18) Grimme, S.; Antony, J.; Ehrlich, S.; Krieg, H. A Consistent and Accurate Ab Initio Parametrization of Density Functional Dispersion Correction (DFT-D) for the 94 Elements H-Pu. *J. Chem. Phys.* **2010**, *132* (15), 154104. https://doi.org/10.1063/1.3382344.
(19) Ong, S. P.; Richards, W. D.; Jain, A.; Hautier, G.; Kocher, M.; Cholia, S.; Gunter, D.; Chevrier, V. L.; Persson, K. A.; Ceder, G. Python Materials Genomics (Pymatgen): A Robust, Open-Source Python Library for Materials Analysis. *Comput. Mater. Sci.* **2013**, *68*, 314–319. https://doi.org/10.1016/j.commatsci.2012.10.028.
